# Discovering cell-active BCL6 inhibitors: effectively combining biochemical HTS with multiple biophysical techniques, X-ray crystallography and cell-based assays

**DOI:** 10.1038/s41598-022-23264-z

**Published:** 2022-11-03

**Authors:** Olivier A. Pierrat, Manjuan Liu, Gavin W. Collie, Kartika Shetty, Matthew J. Rodrigues, Yann-Vaï Le Bihan, Emma A. Gunnell, P. Craig McAndrew, Mark Stubbs, Martin G. Rowlands, Norhakim Yahya, Erald Shehu, Rachel Talbot, Lisa Pickard, Benjamin R. Bellenie, Kwai-Ming J. Cheung, Ludovic Drouin, Paolo Innocenti, Hannah Woodward, Owen A. Davis, Matthew G. Lloyd, Ana Varela, Rosemary Huckvale, Fabio Broccatelli, Michael Carter, David Galiwango, Angela Hayes, Florence I. Raynaud, Christopher Bryant, Steven Whittaker, Olivia W. Rossanese, Swen Hoelder, Rosemary Burke, Rob L. M. van Montfort

**Affiliations:** 1 Division of Cancer Therapeutics, Centre for Cancer Drug Discovery, The Institute of Cancer Research, London, SM2 5NG UK; 2 Division of Structural Biology, The Institute of Cancer Research, London, SW3 6JB UK

**Keywords:** Biophysical chemistry, Proteins, Structural biology, Cancer, Oncology, Drug discovery, Biochemistry

## Abstract

By suppressing gene transcription through the recruitment of corepressor proteins, B-cell lymphoma 6 (BCL6) protein controls a transcriptional network required for the formation and maintenance of B-cell germinal centres. As BCL6 deregulation is implicated in the development of Diffuse Large B-Cell Lymphoma, we sought to discover novel small molecule inhibitors that disrupt the BCL6-corepressor protein–protein interaction (PPI). Here we report our hit finding and compound optimisation strategies, which provide insight into the multi-faceted orthogonal approaches that are needed to tackle this challenging PPI with small molecule inhibitors. Using a 1536-well plate fluorescence polarisation high throughput screen we identified multiple hit series, which were followed up by hit confirmation using a thermal shift assay, surface plasmon resonance and ligand-observed NMR. We determined X-ray structures of BCL6 bound to compounds from nine different series, enabling a structure-based drug design approach to improve their weak biochemical potency. We developed a time-resolved fluorescence energy transfer biochemical assay and a nano bioluminescence resonance energy transfer cellular assay to monitor cellular activity during compound optimisation. This workflow led to the discovery of novel inhibitors with respective biochemical and cellular potencies (IC_50s_) in the sub-micromolar and low micromolar range.

## Introduction

B-cell lymphoma 6 (BCL6) protein, a member of the Broad-Complex, Tramtrack and Bric-à-brac (BTB) / Pox virus and Zinc finger (POZ) family of transcription factors, plays a key role in the formation and maintenance of B-cell germinal centres^1^. During normal humoral immune response, BCL6 is upregulated to enable the production of high affinity antibodies. Its function is then terminated by its downregulation and the disruption of BCL6 transcriptional complexes. In Diffuse Large B-cell Lymphoma (DLBCL), however, the aberrant persistence of BCL6 expression (e.g., due to chromosome translocations and/or point mutations) plays a critical role in lymphomagenesis^2–6^.

BCL6 has an *N-*terminal BTB domain, responsible for obligate homodimerisation; a long unstructured central region involved in interaction with the MTA3/NuRD complex; and six *C*-terminal C2H2-type Zinc-fingers involved in DNA binding^7,8^. To repress transcription of its target genes, BCL6 interacts with various protein complexes^6^ and recruits corepressor proteins such as BCOR, NCOR, or SMRT (also called NCOR2) to its *N-*terminal BTB domain dimer in a mutually exclusive manner^9^. NCOR and SMRT form similar chromatin-modifying complexes that deacetylate histones^10,11^, whereas BCOR forms a variant Polycomb PRC1 complex which exerts multiple distinct effects on chromatin^12^. Disruption of the SMRT or BCOR corepressor interaction with the BCL6 BTB domain is sufficient to inhibit DLBCL cell growth^9,13^. Consequently, we and others have sought to discover small molecule inhibitors that disrupt these interactions, aiming at identifying new treatments for BCL6-driven lymphomas, and multiple small molecule inhibitors have now been reported for BCL6^14–24^.

Here we report our BCL6 hit finding, validation, and optimisation campaign to provide insights into the multi-faceted and orthogonal approaches needed to tackle a challenging protein–protein interaction (PPI) with small molecule inhibitors, particularly when validated tool molecules are lacking at the start of the process. Using a fluorescence polarisation (FP) assay in a high throughput screen (HTS) for hit identification and multiple orthogonal biophysical techniques, including a thermal shift assay (TSA), surface plasmon resonance (SPR) and ligand-observed nuclear magnetic resonance (LO-NMR) for hit validation, we identified nine novel and chemically diverse series of inhibitors of the BCL6 BTB-corepressor interaction. We established a robust, soakable, high-resolution BCL6 BTB domain crystal system which allowed us to elucidate the binding mode of all nine hit series and map the binding site hot spots in relation to the binding of the corepressor peptides. For hit optimisation, a time-resolved fluorescence resonance energy transfer (TR-FRET) was developed to provide an assay with greater sensitivity for the determination of inhibitor potencies significantly below the tight binding limit of the original FP assay. To determine the cellular activity of improved inhibitors, we established two assays; a BCL6 InCell Hunter™ assay (DiscoverX/Eurofins) to confirm target engagement, and a Nano Bioluminescence Resonance Energy Transfer (NanoBRET, Promega UK Ltd)^25^ to demonstrate and quantify the inhibition of the full length BCL6/SMRT protein–protein interaction in cells. With this comprehensive assay cascade, combined with a structure-based drug design approach, we improved the weak potency of the primary hits and generated benzimidazolone-based inhibitors with sub-micromolar biochemical IC_50s_, which translated into low micromolar IC_50s_ in the NanoBRET cell-based assay.

## Results and discussion

### High throughput screen

For the HTS, we used a FP competition assay that monitored the disruption of the interaction of BCL6 BTB domain with a fluorescently labelled BCOR peptide^9^. This technology was chosen as it was easy to set up and amenable to miniaturisation into a 1536-well plate format. Key enablers were the ability to produce large amounts of stable recombinant BCL6 BTB domain (20–35 mg of pure protein obtained per litre of cell culture) and a high quality stable 17 amino acid Alexa Fluor 633-conjugated BCOR peptide probe with a FP K_D_ of 1.7 ± 0.3 µM (Figure S1A). At the time of the screen, only one weak small molecule inhibitor, 79–6, had been reported^17^. Therefore, unlabelled BCOR and SMRT peptides were tested for their suitability as positive controls in the FP assay. The BCOR and SMRT peptides gave average FP IC_50_ values of 13 ± 1 µM and 16 ± 6 µM, respectively (Fig. S1B). As the BCOR peptide was the most stable of these two peptides, it was selected as a positive control peptide.

**Figure 1:**
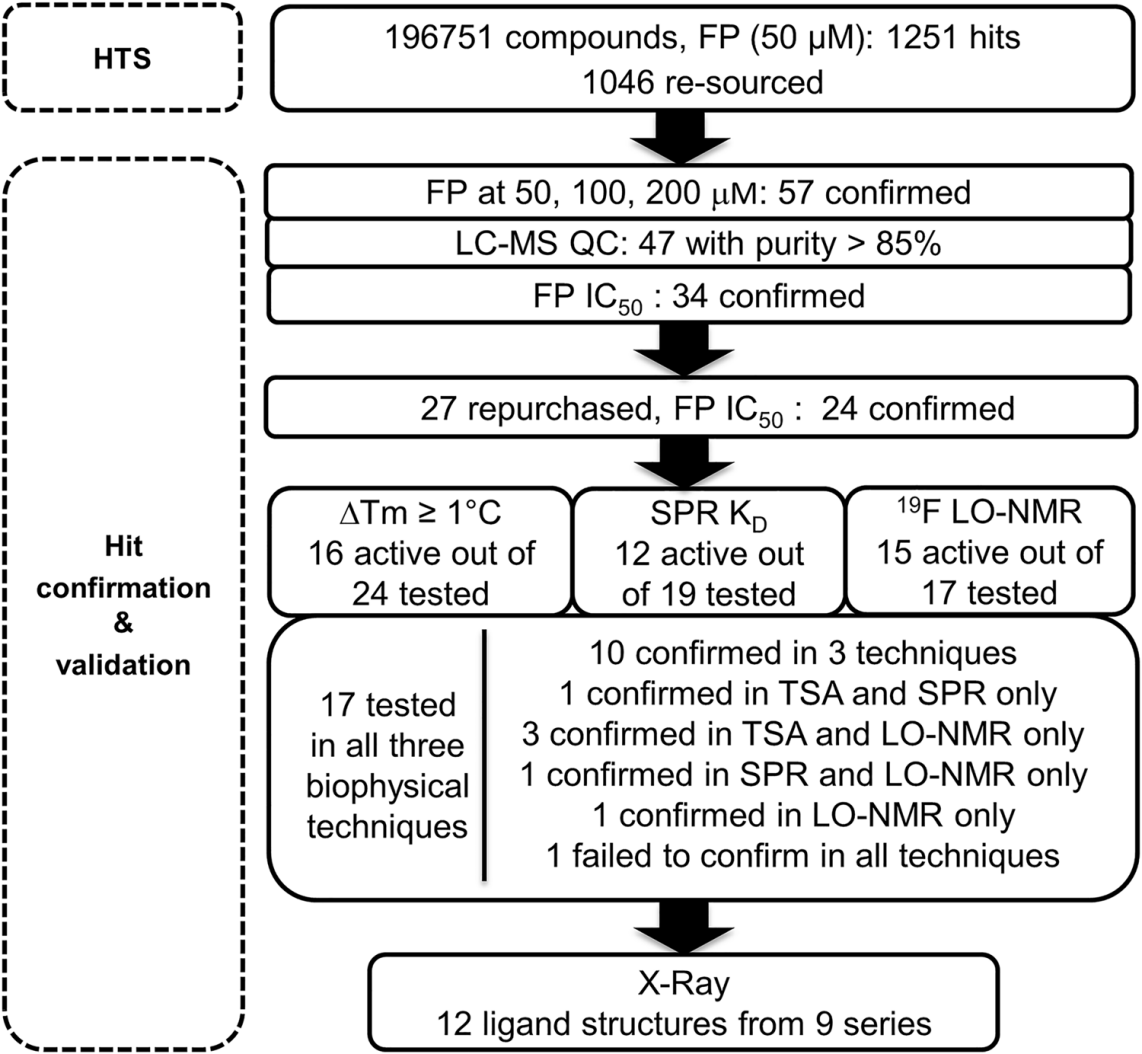
Summary of HTS campaign and hit validation by orthogonal biophysical techniques. An HTS was carried out using an FP assay with an FP-probe based on a BCOR corepressor peptide. The primary HTS hits were subjected to a first confirmation step at three concentrations in the FP assay. Hits confirmed in this step were progressed to IC_50_ determination in the FP assay using repurchased material. Active hits were characterised in orthogonal biophysical techniques including TSA, SPR and LO-NMR using a ^19^F-labelled WVIP reporter peptide. Compound selection for the respective biophysical assays was heavily based on experimental aqueous solubility experiments on the confirmed hits using NMR and HPLC. Hits confirmed to bind the BCL6-BTB domain in one or more biophysical methods were selected for binding mode elucidation by X-ray crystallography which yielded 12 protein–ligand structures representing 9 different chemical series.

Using the FP assay, we screened an in-house HTS library of 196751 compounds at a 50 µM final concentration. The assay window ranged between 80 and 90 mP with Z’ values varying between 0.6 and 0.8. Compounds were considered hits if they displayed greater than 16% inhibition, representing two standard deviations from the mean value of -2.9%. The slightly negative mean value indicated an uneven Gaussian distribution of the total FP mP values registered during the full HTS, due to the scattering of polarised light produced by insoluble compounds. This low cut-off was chosen to identify the weakly active compounds we were expecting for this challenging PPI target. The screen yielded 1251 primary hits, corresponding to a hit rate of 0.6%. Of these, 100 compounds displayed a total fluorescence intensity greater than 110% of the DMSO control, were consequently flagged as fluorescence interferers, and removed from the hit list^26^. We also removed 89 chemically intractable compounds and 16 compounds that were no longer available, leaving 1046 compounds for a confirmation screen (Fig. 1).

In the confirmation FP screen, the primary hits were tested at concentrations of 50, 100 and 200 µM to confirm their activity and to investigate whether it was concentration dependent. This yielded 57 confirmed hits, 47 of which passed LC–MS QC analysis (purity greater than 85%) and were taken forward for IC_50_ determination. Excluding one fluorescence interferer and twelve compounds displaying concentration response curves with a hill slope greater than 2, fifteen compounds showed a 25–48% inhibition at the maximal concentration of 200 µM and nineteen compounds yielded IC_50_ values between 23 and 197 µM.

In our experience it is crucial to reconfirm compound activity using a different batch of solid material. Seven primary hits were not commercially available, but we repurchased 16 compounds with measurable IC_50_ values and hill slope between 0.5 and 2, along with 11 weakly active compounds structurally related to more active compounds. We hypothesised that retesting these structurally related compounds at higher concentration (> 200 µM) could unravel some initial structure activity relationships. We retested the 27 repurchased hits in the FP assay at variable concentrations up to 2 mM. We reconfirmed 24 compounds, 23 with IC_50_ values ranging from 70 to 700 µM and one weakly active with 34% inhibition at 1 mM (Fig. 1 and Table S1).

### Hit validation in biophysical assays

To further validate our hits as genuine BCL6 inhibitors we used three orthogonal biophysical techniques in parallel, each differing in sensitivity and susceptibility to interference: TSA, SPR and LO-NMR. Additionally, determination of the kinetic solubility of the hits by NMR or HPLC^15^, revealed a compound solubility range from high (≥ 500 µM) to poor (< 30 µM) in aqueous buffer (Table S1). The low solubility of certain hits meant that not all hits could be validated in each technique, which especially affected the SPR and LO-NMR experiments.

#### TSA

The binding of repurchased compounds was investigated using a BCL6 thermal shift assay^27^. The apo-BCL6 BTB domain gave a melting temperature (T_m_) of 56.7 ± 0.1 °C, whilst a positive ΔT_m_ of 6.5 °C was observed in the presence of 200 µM of the BCOR control peptide. We tested the 24 hits reconfirmed in the FP assay at 200 and 400 µM and identified 16 compounds with a positive ΔT_m_ between + 1 and + 3.5 °C (Fig. 1 and Table S1) and 8 with no or a very small shift (ΔT_m_ = 0.0 ± 0.5 °C).

#### SPR

**Figure 2:**
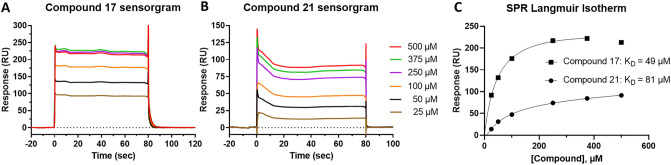
SPR results for the two hits showing the highest affinity for the BCL6 BTB dimer. (**A**) Sensorgrams of compound **17** at different concentrations. (**B**) Sensorgrams of compound **21** at different concentrations. (**C**) Corresponding binding curves and calculated K_Ds_ for compounds **17** and **21**.

Using SPR, which allows measurement of the equilibrium binding constant (K_D_) even for weakly bound small molecules and fragments^20,28^, we tested 19 of the 24 reconfirmed hits for direct binding to the BCL6 BTB domain. Four hits were not assayed because of their low solubility (< 30 µM) and one because of its low FP activity. Of the 19 hits tested, 7 failed to give measurable K_D_ values due to unsaturating binding curves, poor quality sensorgrams, or a binding stoichiometry inconsistent with a 1:1 binding model (Experimental/Theoretical R_max_ ratio below 0.5 or above 3). We confirmed the binding of 12 compounds with K_D_ values ranging from 49 to 1 mM (Fig. 1 and Table S1). The two strongest binders were compounds **17** and **21** with respective K_Ds_ of 49 µM and 81 µM (Fig. 2 and Table S1).

#### LO-NMR

In our experience, and that of other research groups, LO-NMR experiments are most informative if set-up in a competition format with a tight binding control compound or a weakly binding reporter molecule^29^. To further validate the reconfirmed HTS hits, we sought to identify a small peptide with low affinity for the BCL6 BTB domain that could be used as a reporter probe in a LO-NMR displacement assay. We tested the binding of small peptides of 4 to 8 amino-acids with sequences based on the BCOR or the SMRT BCL6 binding domains^9,30^ using ^1^H-NMR (Figure S2A). We identified the tetrapeptide WVVP, based on the BCOR peptide sequence, as the smallest peptide fragment which retained a BCL6 BTB domain binding affinity detectable by ^1^H-NMR (Fig. S2A,C). We then tested a set of short peptides based on the WVVP peptide and showed that the hybrid BCOR/SMRT WVIP peptide gave the highest peak reduction in the presence of the BCL6 BTB domain (Fig. S2B,D). In addition, the ^1^H-NMR signals of the WVVP and WVIP peptides recovered in the presence of a 17 residue BCOR peptide (Fig. S2C,D), confirming that the binding sites of the two tetrapeptides overlap with the BCOR corepressor binding site. The WVIP peptide was selected as the reporter probe for our NMR displacement assay as it combines the smallest sequence with the largest peak reduction (Fig. S2B,D).

**Figure 3:**
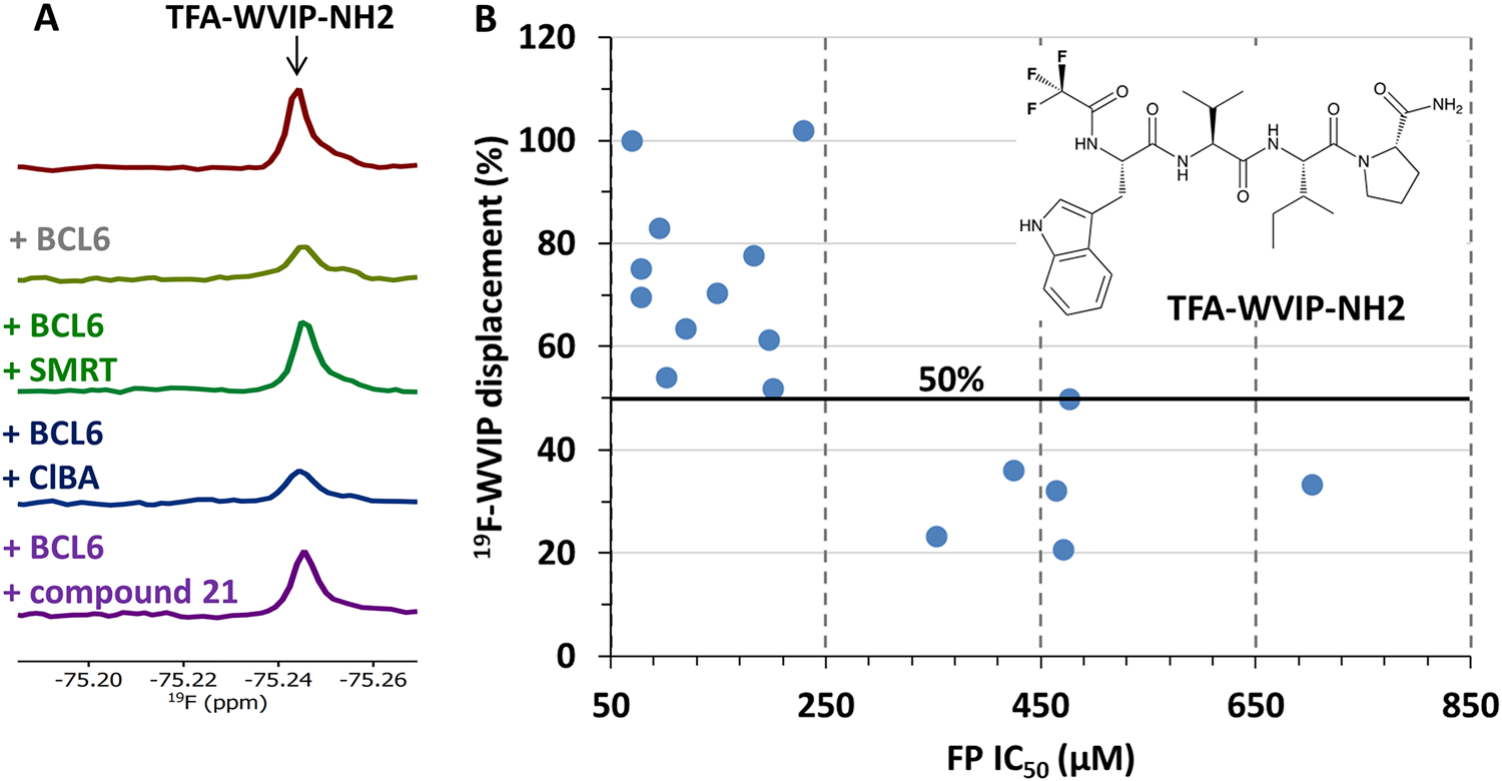
Hit validation in ^19^F Ligand-Observed NMR. The TFA-WVIP-NH_2_ tetrapeptide was used as a reporter in a ^19^F-LO-NMR peptide displacement assay. (**A**) The SMRT peptide and compound **21** (bright green and purple traces), but not the negative control 4-chlorobenzoic acid (ClBA, blue trace), were able to displace the BCL6-bound TFA-WVIP-NH_2_ peptide, as indicated by the recovery of the peptide fluorine signal. (**B**) The LO-NMR data correlated well with biochemical FP potency, with the 11 strongest FP hits yielding a peptide displacement greater than 50% in LO-NMR.

To prevent signal overlap in ^1^H-NMR experiments, we synthesized a WVIP peptide fluorinated (trifluoro acetic acid, TFA) at the *N-*terminus and amidated at the *C*-terminus (TFA-WVIP-NH_2_, Fig. 3B) to use as a reporter in T_2_ relaxation edited ^19^F-NMR experiments. As illustrated in Fig. 3A, the peak of the TFA-WVIP-NH_2_ tetrapeptide was reduced upon binding to the BCL6 BTB domain but recovered upon competition with the SMRT control peptide and with compound **21**, one of our exemplar hits, but not in the presence of the negative control 4-chlorobenzoic acid. We tested 17 of the 24 confirmed HTS hits in the ^19^F-NMR reporter assay. The 7 remaining compounds were not characterised because of their low solubility (< 30 µM), low signal-to-noise, or because representative compounds of the same series were assayed. The 11 compounds with the strongest ^19^F-NMR displacements corresponded to hits with the highest potency (IC_50_ < 250 µM) in the FP assay (Fig. 3B), while the 6 compounds with lower potency (IC_50_ > 350 µM) showed the weakest displacements (≤ 50%). This correlation increased our confidence in the 11 most potent hits.

In summary, of the 24 primary hits confirmed in the FP assay, 17 were tested across all three biophysical techniques, and 16 were validated in one or more of these orthogonal assays (Fig. 1 and Table S1). The validated hits were clustered in five chemical classes (triazolo-pyrimidine, cyano-trifluoromethyl-pyridine, thiazole, pyrimidine-benzimidazolone and pyrazolo-pyrimidinone) and four additional singletons (Table S1). Altogether, the evaluation of HTS hits by multiple biophysical approaches gave us a high confidence in our validated hit list and encouraged us to attempt elucidating their binding mode by X-ray crystallography.

### Binding-mode determination by X-ray crystallography

**Figure 4:**
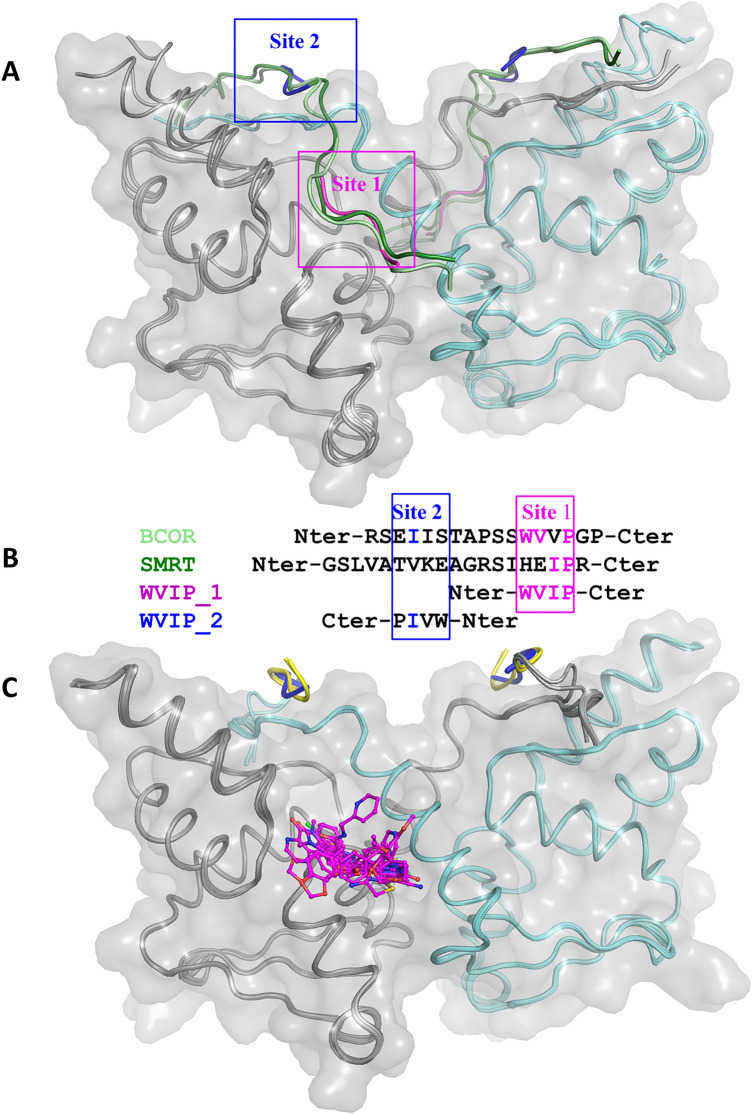
Natural corepressors, WVIP peptide, and HTS hits bind to the same area on the surface of BCL6 BTB domain dimer. (**A**) Overlay of the BCL6 BTB domain dimer bound to the SMRT corepressor peptide (dark green, PDB ID 1R2B), the BCOR corepressor peptide (light green, PDB ID 3BIM) and the WVIP peptide bound at site 1 (magenta) and site 2 (blue). The BCL6 dimer is shown in ribbon representation with the two individual monomers coloured in grey and cyan with a grey semi-transparent surface superimposed. (**B**) Sequence alignment reflecting the structural overlay of the corepressors and the two molecules of the WVIP peptide bound at site 1 and site 2, respectively. (**C**) Overlay of BCL6 BTB domain dimer bound to the 12 structurally confirmed HTS hits (magenta). The colour scheme and surface representation of the BCL6 dimer are the same as in panel (**A**). The HTS hits are shown as magenta ball and sticks. The *N*-terminal uncleaved TEV sequence present in the Flag-TEV-BCL6 construct, used for some of the BCL6-inhibitor structures and binding at the second WVIP binding site (site 2), is shown as a yellow ribbon.

Our first attempts to obtain a soakable BCL6 BTB domain crystal system only yielded weakly diffracting crystals. However, when we crystallised the BCL6 BTB domain in the presence of the WVIP tetrapeptide identified by LO-NMR, we obtained strongly diffracting crystals and determined the WVIP-bound BCL6 BTB crystal structure at 2.05 Å resolution (Table S2). The WVIP tetrapeptide binds in two locations in the BCL6 BTB peptide binding groove. The primary WVIP binding site (Site 1 in Fig. 4) overlaps with the corresponding residues in the BCOR and SMRT peptides^9,30^, and its binding mode is like that of these longer peptides (Fig. S3). The interaction of BCL6 with the WVIP peptide is mainly driven by polar interactions. These include H-bonds between the carbonyl group of Met51 in BCL6 and the side chain NH-group of the peptide’s tryptophan residue, the side chains of Arg24 and Arg28 in BCL6 and two carbonyls of the peptide’s main chain, and between the side chain of Asn21 in BCL6 and a carbonyl and an NH-group in the main chain of the WVIP peptide (Fig. S3C). Hydrophobic interactions further stabilise the peptide. These include an interaction between the side chain of the peptide’s tryptophan residue and the main chain of BCL6 residues 53–55, and an intercalation of the side chain of the peptide’s isoleucine in a small hydrophobic pocket between Tyr58 and Asn21 of BCL6 (Fig. S3C). In the second site, the WVIP peptide binds in a reversed orientation as compared to the corepressors (Site 2 in Fig. 4A,B). As this second WVIP molecule is involved in extensive crystal contacts with a neighbouring BCL6 molecule, stabilising the crystal packing, this binding event is most likely a crystallisation artefact.

Consistent with the low affinity observed in the FP assay (16% inhibition at 980 µM, n = 4, Fig. S3D), the WVIP peptide bound to site 1 could be easily displaced in soaking experiments with the HTS hits. Based on the results from the biophysical hit confirmation experiments, we selected 13 validated hits, representing the five different chemical series and three singletons, for structural characterisation using X-ray crystallography. We solved the crystal structures of the BCL6 BTB domain bound to 9 different hits at a resolution ranging between 1.38 and 1.85 Å (Table S2, Fig. 4C and S4), which corresponds to a 69% success rate.

Based on publications from Takeda Pharmaceutical^23,31^ we implemented a second BCL6 BTB domain crystal system which did not require the addition of a peptide for crystallisation. In these crystals, an uncleaved *N*-terminal Flag-TEV tag replaced the WVIP peptide bound to site 2, stabilising the crystal packing, but leaving site 1 unoccluded (Fig. 4C). Using this crystal system, we solved crystal structures of the BCL6 BTB domain bound to 3 additional hits, increasing the success rate for the structural characterisation of validated HTS hits to 92% (Table S2, Fig. 4C and S4).

As expected, in all 12 ligand-bound BCL6 structures the compound binds in site 1 (Fig. 4C), which is consistent with the competition with the peptide-based probes in the LO-NMR and FP assays. Despite their low chemical similarity, the different compounds all bind to BCL6 via similar interactions, the vast majority mimicking the interactions of BCL6 with its corepressors SMRT and BCOR (Fig. S3, S4). Importantly, all compounds, except the cyano-trifluoromethyl pyridines **11** and **13**, interact via an H-bond between an NH group and the main chain carbonyl of Met51 in BCL6 similar to the interaction formed by the Trp509 and His1426 side chains in the BCOR and SMRT corepressor peptides^9,30^. Additionally, all compounds intercalate between the side chains of Tyr58 and Asn21, as observed for Ile1428 in SMRT and Val511 in BCOR. Furthermore, all compounds except **11** have a substituent that stacks against the main chain of BCL6 residues 53–55, mimicking the interactions of the side chains of His1426 and Trp509 in the SMRT and BCOR peptides. Several compounds formed additional H-bonds to BCL6 residues further mimicking interactions observed in the BCL6 corepressors structures. For example, the benzimidazolone moieties in **17** and **18**, the thiazole-amide in **14**, and the pteridinone in **22** form direct and/or water-mediated H-bonds with main chain atoms of Glu115 and/or His116, like the interactions of the side chain of Ser508 in the BCOR peptide. In addition, **17**, **2**, **11**, **14** and **15** all mimic interactions with main chain atoms from both corepressors by forming H-bond networks (direct or water-mediated) with one or more of the following BCL6 residues: Asn21, Arg24 or Arg28. Several of the BCL6-ligand interactions observed for our HTS hits, such as the intercalation between Tyr58 and Asn21, the key H-bond to Met51, the H-bond to Glu115, and the interactions with Arg24 and Arg28, are also present in reported BCL6 inhibitors with different chemical scaffolds binding in the same hotspot^17,18,20–24,32,33^, which is not surprising as they all mimic the interactions observed with the SMRT and BCOR corepressors.

In conclusion, structural characterisation revealed the key BCL6 binding interactions of our HTS hits, which proved crucial in enabling a structure-based drug design approach to generate more potent inhibitors with drug-like physicochemical properties for progression into cellular activity assays.

### TR-FRET assay

**Figure 5:**
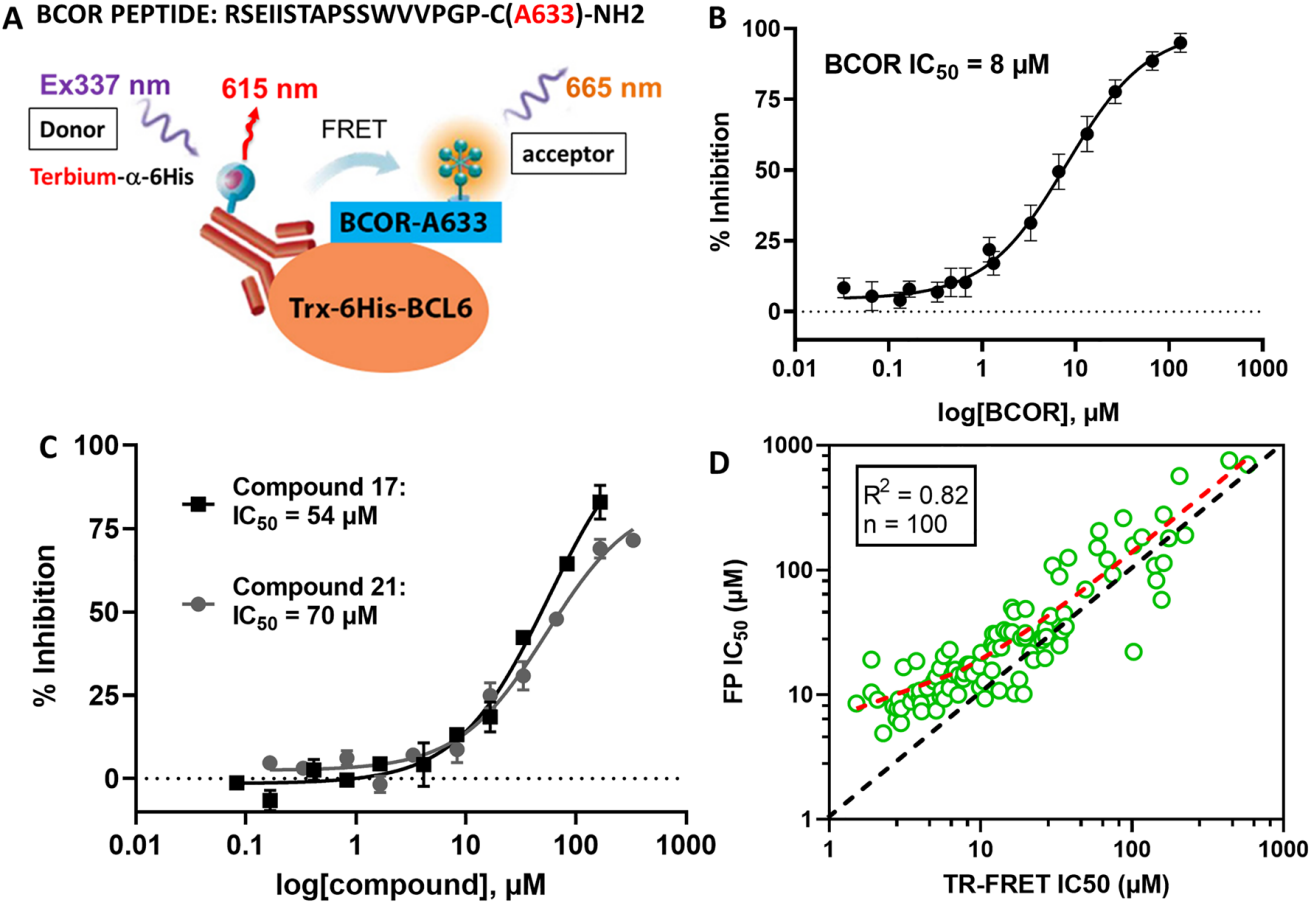
TR-FRET assay development and correlation with the FP assay. (**A**) Schematic representation of the BCL6 BTB – BCOR peptide interaction as measured by TR-FRET assay format. (**B**) Unlabelled BCOR peptide competed with the Alexa-633 conjugated BCOR peptide in the TR-FRET reaction with an IC_50_ of 8 ± 0.8 µM, n = 20. (**C**) The two most potent HTS hits compound **17** and **21** showed respective IC_50_ values of 54 and 70 µM in the TR-FRET assay. (**D**) Correlation between the TR-FRET (x-axis) and the FP (y-axis) assays for the benzimidazolone series of BCL6 inhibitors. The BCL6 BTB concentration was 3 µM in the FP and 10 nM in the TR-FRET assay. The two biochemical assays correlated with a R^2^ of 0.82, but most compounds displayed higher potencies in the TR-FRET assay compared to the FP assay (red dotted trendline above the black dotted unity line). As compound potencies approached the tight binding limit of the FP assay (IC_50_ < 5 µM), the correlation between the two assays decreased.

Our hit optimisation efforts focused on inhibitors obtained by merging the cyano-chloro-pyridine in **21** and the benzimidazolone moiety in **17**^15^, as these were among the most potent HTS hits with chemically tractable scaffolds (Fig. 2 and 5C, Table S1). This strategy led to a significant improvement of the biochemical potency of our compounds. However, during hit optimisation it became apparent that the sensitivity of the FP assay was limited by the weak affinity of the probe for the BCL6 BTB domain (Fig. S1A) and the large amount of protein (3 µM) necessary to generate a robust FP signal. As the range of resolvable inhibitor potencies in an FP assay is limited by the affinity of the fluorescent ligand^34^, we developed a semi-direct TR-FRET assay based on a Thioredoxin-6His-tagged BCL6 BTB variant and the fluorescent-labelled BCOR-A633 FP probe (Fig. 5A). In the FP-assay, the K_D_ of the Thioredoxin-6His-tagged BCL6 BTB protein interaction with the BCOR-A633 peptide was measured as 1.74 µM, very similar to the K_D_ of the untagged BCL6 BTB domain (Fig. S1A). In the new TR-FRET assay, Z’ values ranging from 0.7 to 0.9 and an assay window of up to 2.5 were obtained routinely with as little as 1 nM protein, thus achieving a very robust and sensitive assay format. The protein-probe interaction could be disrupted with unlabelled BCOR peptide (Fig. 5B, IC_50_ = 8.0 ± 0.8 µM) and compounds **17** and **21** (Fig. 5C, respective IC_50_ of 54 and 70 µM). The HTS hits and their analogues showed a good correlation between the FP and TR-FRET assay formats (Fig. 5D, R^2^ = 0.82, n = 100, 1 µM < TR-FRET IC_50_ < 600 µM). However, later in the hit optimisation stage, when the compound potency reached the low micromolar range (TR-FRET IC_50_ < 5, Fig. 5D), the predicted tight binding limit of the FP assay, TR-FRET IC_50_ values became discriminatory and allowed robust characterisation of BCL6 inhibitors with sub-micromolar biochemical IC_50s_ (Table 1)^15^.

**Table 1:** Biochemical and cellular activity of benzimidazolones at early-stage optimisation.

Structure	No	HPLC Sol. (µM)^a^	TR-FRET Geo Mean IC_50_ (µM)^b^	TR-FRET N	TR-FRET pIC_50_ mean	TR-FRET pIC_50_ SD	Nano BRET Geo Mean IC_50_ (µM)^c^	Nano BRET N	Nano BRET pIC_50_ mean	Nano BRET pIC_50_ SD	PAMPA pH7.4 (cm.s^-1^)
	36	16	3.11	5	5.51	0.29	33.5	4	4.48	0.08	48
	37	25	0.86	3	6.07	0.03	7.72	3	5.11	0.11	6
	27	80	0.50	3	6.30	0.07	2.93	4	5.53	0.24	19

^a^Aqueous solubility measured by HPLC in PBS buffer pH 7.4 and 1% DMSO, at maximum targeted concentration of 100 µM.

^b^TR-FRET measured in presence of 1 nM BCL6 BTB domain. Data represents the geometric mean from n = 3–5 replicates.

^c^NanoBRET assay using configuration illustrated in Fig. 7C. Data represents the geometric mean from n = 3–4 replicates.

### Cellular assays

We developed cell-based assays to test the translation of the improved biochemical potency of our most advanced compound series into cellular activity. We established two cell-based assays: an InCELL Hunter™ target engagement assay (Eurofins-DiscoverX) and a NanoBRET assay (Promega UK Ltd) technologies.

**Figure 6:**
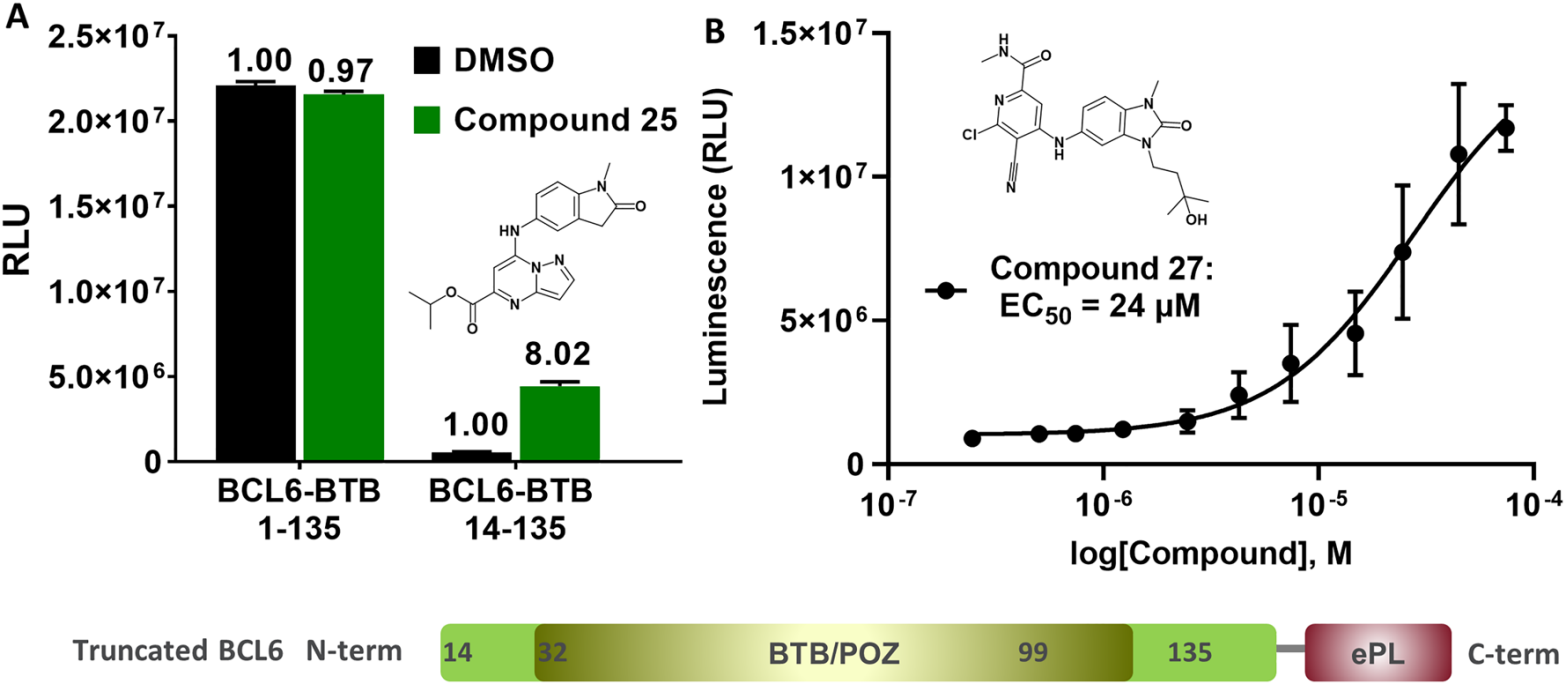
InCELL Hunter™ cellular target engagement assay. (**A**) HEK293T cells, transfected with DNA plasmids expressing complete *C*-terminally ePL-tagged BCL6 BTB (amino-acids 1–135) or truncated BCL6 BTB (amino-acids 14–135), were incubated with the oxindole compound **25** (30 µM) for 6 h before luminescence was detected on a plate reader (error bars represents standard deviation from n = 3); (**B**) HEK293T cells, transfected with *C*-terminally ePL-tagged truncated BCL6 BTB 14–135, were incubated with increasing concentrations of the benzimidazolone compound **27** for 6 h before luminescence detection (EC_50_ 24 ± 9 µM, error bars represents standard deviation from n = 3).

The InCELL Hunter™ assay is based upon the measurement of compound-mediated stabilisation of an enhanced ProLabel (ePL) tagged version of the protein of interest in cells^35–38^. A prerequisite for success was to identify a control compound known to bind to the BCL6-BTB dimer with properties commensurate with cell permeability. To set up and validate the assay we synthesized the oxindole-based inhibitor compound **25** (Fig. 6A), which is similar to published BCL6 oxindole-based inhibitors^20^. Compound **25** has a TR-FRET IC_50_ value of 2.1 ± 0.1 µM (n = 2), a high passive cell permeability of 110.10^–6^ cm/sec at pH 7.4 in the parallel artificial membrane permeability assay (PAMPA) and a low Caco-2 efflux ratio of 0.72.

Using a BCL6 construct comprising the complete BCL6 BTB domain (amino acids 1–135) proved unsuccessful, but we obtained an acceptable assay window using an *N*-terminally truncated BCL6 BTB domain (amino acids 14–135) fused to a *C*-terminal ePL tag. This construct allowed us to confirm BCL6 target engagement of compound **25** in HEK293T cells (Fig. 6A). Subsequently, we demonstrated BCL6 target engagement for 10 compounds from our benzimidazole series with EC_50_ values ranging from 5 to 75 µM (Table S3), exemplified by compound **27**^14^ (Fig. 6B).

**Figure 7:**
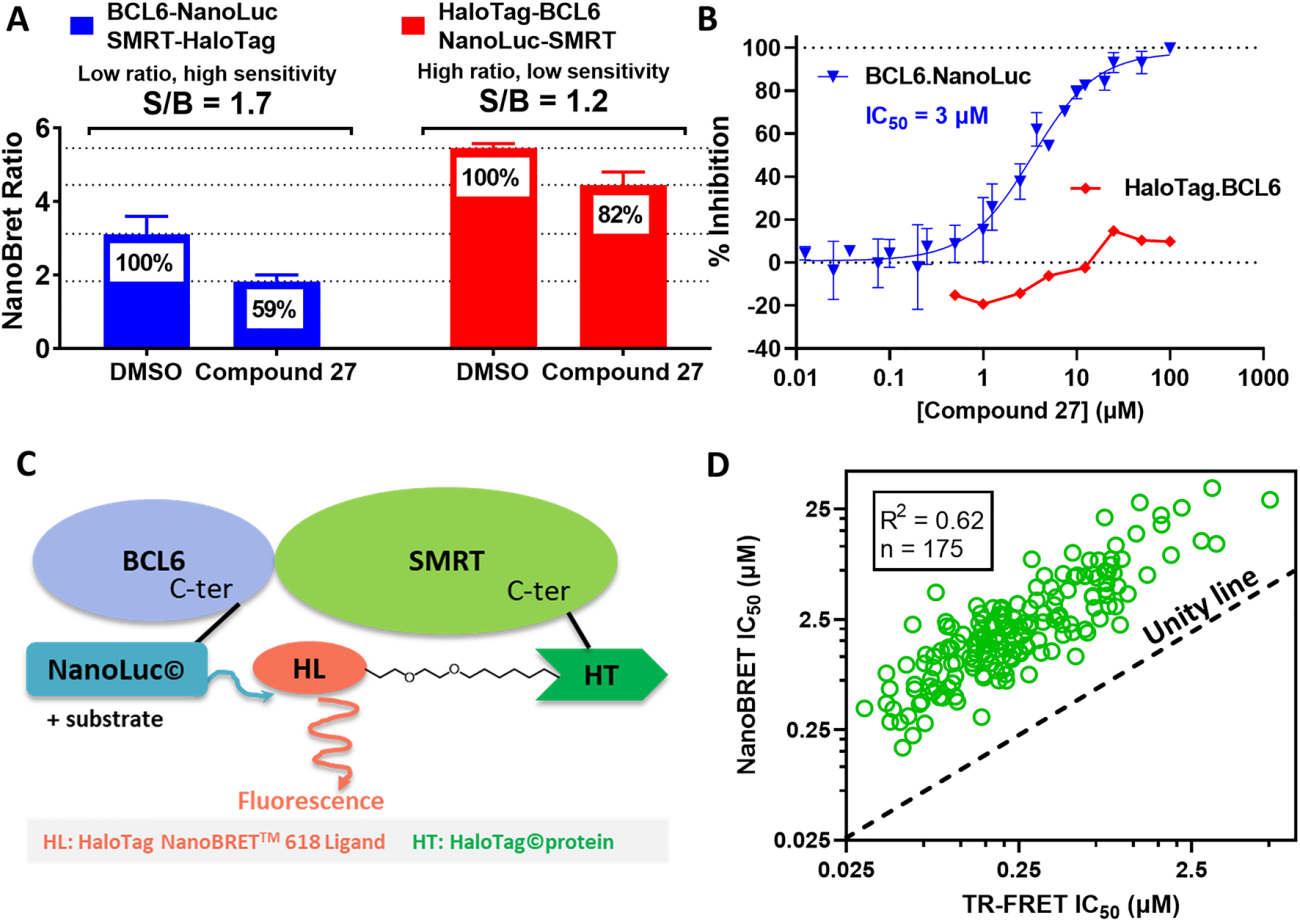
NanoBRET assay sensitivity and activity of benzimidazolone compounds. (**A**) Effect of the NanoBRET configuration on the assay sensitivity to compound inhibition in HEK293T cells. The blue histograms show the NanoBRET ratio for the *C*-terminally NanoLuc tagged BCL6 and HaloTag tagged SMRT with and without the BCL6 inhibitor **27**. The red histograms show the ratio for the *N*-terminally HaloTag tagged BCL6 and NanoLuc tagged SMRT with and without **27**. We selected the former because of its higher sensitivity to compound inhibition after 6 h treatment with 25 µM **27**. (**B**) Concentration responses for **27** in both NanoBRET configurations. An IC_50_ value could be determined with *C*-terminally NanoLuc tagged BCL6 (blue triangles), but not when BCL6 was tagged *N*-terminally with the Halo-Tag (red diamonds). (**C**) Schematic representation of the optimal NanoBRET configuration for maximal sensitivity to compound inhibition. (**D**) Plot showing the correlation (R^2^ = 0.62, n = 175) between biochemical (TR-FRET IC_50_ on x-axis) and cellular (NanoBRET IC_50_ on y-axis) activities of inhibitors from the benzimidazolone series. Data points are above the unity line (black), indicating cellular activities were on average an order of magnitude lower than biochemical potencies.

Encouraged by the cellular BCL6 target engagement of our benzimidazolone compounds, we developed a NanoBRET assay (Promega UK Ltd) measuring inhibition of the full-length BCL6/SMRT protein–protein interaction in HEK293T cells, as a more sensitive readout to follow compound structure–activity relationship (SAR). The NanoBRET assay is based on tagged versions of the interacting proteins, one with a NanoLuciferase donor (NanoLuc), the other with an acceptor fluorescent dye (HaloTag + 618 ligand), which produces a bioluminescence resonance energy transfer (BRET) signal when the two proteins are near each other^25,39^. Since the donor and acceptor can each be fused to the N- or *C*-terminus of BCL6 and SMRT, eight possible BCL6/SMRT BRET configurations were tested (Fig. S5A-C). To characterise compound inhibition, we selected two configurations with high BRET ratios and donor signals, one with BCL6 *N*-terminally fused to the HaloTag (HaloTag.BCL6/NanoLuc.SMRT), the other with BCL6 *C*-terminally fused to the NanoLuc (BCL6.NanoLuc/SMRT.HaloTag, Fig. 7A and S5). With the HaloTag.BCL6/NanoLuc.SMRT combination, we observed only very weak inhibition for our inhibitors, as exemplified for compound **27** (Fig. 7A,B). However, using the BCL6.NanoLuc/SMRT.HaloTag configuration (Fig. 7C), we detected compound inhibition with measurable IC_50s_, as also demonstrated by compound **27** (Fig. 7A,B).

Table 1 summarizes the cellular and biochemical activities of three benzimidazolone inhibitors^14,15^ at the early hit optimisation stage. Upon further optimisation, we observed a progressive increase in cellular potency that correlated with the TR-FRET biochemical activity, confirming the suitability of this assay (R^2^ = 0.62, n = 175, Fig. 7D) for optimisation of cellular potency. Consistent with their cellular activity, most benzimidazolones displayed a high cell permeability (75% with PAMPA cell permeability > 20 × 10^6^ cm/sec at pH 7.4) and solubility (67% with NMR kinetic solubility > 150 µM). Altogether, these results demonstrate the robustness of the assay cascade and the quality of our chemical series. Combined with the ability to obtain high-resolution inhibitor-bound BCL6-BTB structures, this enabled us to efficiently progress our drug discovery programme.

## Conclusions

We performed a successful HTS campaign using a FP assay to discover small molecules able to disrupt the BCL6 BTB/BCOR peptide interaction. Despite a low confirmation rate, multiple primary hits with different chemical scaffolds were identified, repurchased, and validated in three orthogonal biophysical techniques (TSA, SPR and LO-NMR). For example, mapping out the long co-repressor binding groove on the BCL6 BTB domain by screening for the binding of smaller peptides using LO-NMR resulted in the identification of the WVIP peptide and its fluorinated version TFA-WVIP. The latter was successfully used in LO-NMR competition experiments to validate the binding of the HTS hits, showing a good correlation with the FP assay.

Confirmation of the binding of the WVIP peptide using X-ray crystallography provided further evidence that compounds showing competition with WVIP in LO-NMR were likely binding in the co-repressor binding groove. Additionally, the WVIP BCL6-BTB co-crystals provided a reliable high resolution crystal system for a detailed binding mode characterisation of exemplars from all hit series and selected singletons. The ligand-bound BCL6 structures showed that our chemically diverse hit series efficiently mimicked key interactions between the natural corepressors BCOR and SMRT and BCL6. Moreover, the WVIP BCL6-BTB co-crystal system, and additional FLAG-TEV-BCL6 crystal system, allowed us to determine the high resolution-structures of the BCL6-BTB domain bound to our improved BCL6 inhibitors in a timely manner, enabling an efficient structure-based drug design approach and significantly facilitating interpretation of the SAR.

Another important step in improving our BCL6 inhibitors was the development of the InCELL Hunter and NanoBRET target cellular engagement assays, to robustly confirm their cellular activity and to enable monitoring of cellular SAR. In both assays, we demonstrated the importance of exploring different configurations to maximise assay sensitivity for compound binding and/or inhibition. The InCELL Hunter assay allowed us to show at an early stage that our inhibitors were able to bind to BCL6 in cells. However, the assay uses a truncated BCL6 BTB domain variant and does not measure cellular activity per se. Previously reported BCL6 cellular assays are based on peptides or truncated protein variants in an AlphaLisa or a mammalian yeast-two hybrid format^18,23^, or used a luciferase-based reporter^22^. None of these assay formats measure the inhibition of the interaction between full-length BCL6 and its full-length SMRT corepressor. In contrast, our NanoBRET assay reports the disruption of the interaction between both full-length proteins in a cellular context. Moreover, the throughput of this assay proved sufficient to measure the cellular potency of hundreds of inhibitors (> 800) generated during the optimisation phase of our BCL6 drug discovery project. We observed a good correlation between the biochemical (TR-FRET) and cellular (NanoBRET) potencies for the benzimidazolone series, validating both our assay cascade and the benzimidazolones as genuine inhibitors of the BCL6-corepressor interaction.

In summary, identifying initial hit matter from an HTS against a PPI target, such as the BCL6-corepressor interaction, can be very challenging, because hit rates are typically low and the proportion of false positives can be high. Additionally, the weak potency of genuine hits makes it difficult to distinguish them from false-positive results. By using a combination of biochemical and biophysical assays, followed by structural confirmation using X-ray crystallography, we were able to overcome each hit validation method’s respective limitations and effectively triage hit compounds, identifying several validated and structurally characterised hit series and singletons. The in vitro TR-FRET assay proved instrumental in the optimisation of biochemical potency and the InCell Hunter and NanoBRET assays were crucial in assessing the cellular activity of the compounds, thus aiding the discovery of a series of potent benzimidazolone- and quinolinone-based BCL6 inhibitors showing sub-micromolar cellular activity and antiproliferative effect in the BCL6-dependent lymphoma cell lines OCI-LY1 and SU-DH-L4^15,40^.

## Methods

### BCL6 constructs used for assays and crystallography

A first construct of BCL6 BTB domain, which we named Trx-6His-HRV3C-BCL6, was obtained by sub-cloning the sequence coding for residues 5–129 of human BCL6, corresponding to its BTB domain, into a pET48b vector with *N*-terminal Thioredoxin and 6-Histidine tags, followed by an HRV-3C protease cleavage site. For the TR-FRET assay, the Trx-6His-HRV3C-BCL6 protein construct was used without cleaving the tag, as a 6His was needed to bind to the anti-6His-Terbium antibody. For the other biochemical and biophysical assays and for the crystallography with WVIP peptide and compounds **2**, **7**, **11**, **13**, **15**, **17**, **19**, **21** and **22**, the tag was removed by HRV-3C protease treatment, generating a simpler BCL6 5–129 product.

For crystallography with compounds **10**, **14** and **18**, the construct described above was modified to introduce a Flag Tag and a TEV cleavage site between the HRV3C and BCL6 sequences. This construct is referred to as Flag-TEV-BCL6.

### BCL6 expression and purification

For both plasmid constructs described above, transformed BL21-AI *E. coli* cells were grown in LB media supplemented with 50 mg/L kanamycin at 37 °C until an OD_600 nm_ of 0.6 was reached. Protein expression was then induced by addition of 0.2 mM IPTG and 0.2% (w/v) Arabinose. Expression was carried out at 18 °C for 18 h. Cells were harvested by centrifugation (5500 g for 30 min at 4 °C) and stored at -80 °C.

Cells were re-suspended in a buffer composed of 20 mM Tris pH 8, 250 mM NaCl, 1 mM MgCl_2_, 0.5 mM TCEP and 5% (v/v) glycerol, 1 × complete ULTRA protease inhibitors and 12.5 U/ml Benzonase. Cells were lysed by sonication followed by centrifugation at 21,000 g for 45 min at 4 °C. The supernatant was loaded onto a HisTrap FF column followed by on-column cleavage of the Trx-6His-HRV3C tag by addition of 2 mg of HRV-3C protease. The cleaved BCL6 5–129, or Flag-TEV-BCL6 5–129, was then eluted and purified further by ResourceQ (for Flag-TEV-BCL6 construct only) and gel filtration using a HiLoad 26/60 Superdex75 column in a buffer containing 20 mM HEPES pH 7.5, 250 mM NaCl, 1 mM TCEP and 5% (v/v) glycerol. The final protein was assessed for purity and molar mass by SDS-PAGE and high-resolution mass spectrometry, respectively.

For the uncleaved Trx-6His-HRV3C-BCL6 protein construct, the protein was directly eluted from the HisTrap FF column without HRV-3C treatment and submitted to Superdex75 gel filtration as described above.

### Fluorescence Polarisation

A modified BCOR peptide containing a *C*-terminal cysteine, labelled with Alexa Fluor (AF)633 C5 maleimide (RSEIISTAPSSWVVPGP-Cys(AF633)-amide) was obtained from Cambridge Research Biochemical. For high throughput screening, the assay was miniaturized from 10 µL in Perkin Elmer 384-well black Proxi Plus plates to 4 µL in Labcyte 1536-well black low dead volume LP-0410 plates. Each complete reaction (a modified version from^9,17^) contained 20 mM Hepes/NaOH pH 8.0, 150 mM NaCl, 0.5 mM tris(2-carboxyethyl)phosphine (TCEP), 0.05% (v/v) Tween 20, 10 nM BCOR-AF633 peptide, 3 µM BCL6 BTB and 1–2% (v/v) DMSO. At this protein concentration, we recorded a FP signal of 80 to 90 mP, corresponding to about 55% BCOR-AF633 bound to the BCL6 BTB domain. Compounds and control BCOR peptide were dispensed using an Echo550 acoustic dispenser (Beckman Coulter). Protein and peptide reagents were added using a Multidrop combi (Thermo Scientific) or a Tempest v2 (Formulatrix). After 2 h incubation at room temperature, plates were read on an Envision plate reader (Perkin Elmer) with excitation and emission wavelengths of 620 nm and 688 nm, respectively, or Pherastar FSX (BMG Labtech) with FP filters module 590-50/675-50/675-50. To determine the % inhibition from raw mP values using Dotmatics or Prism (GraphPad Software, La Jolla, CA), test wells were normalised to control wells containing either DMSO or 100 µM unlabelled BCOR competitor peptide. As a low signal control, we used either the maximal inhibition by BCOR in the HTS campaign, or the signal obtained from the probe in the absence of BCL6 for IC_50_ determination. IC_50_ values were calculated using a sigmoidal dose–response (variable slope) four- parameter equation. Beside the FP measurement, the total fluorescence intensity (TFI) was recorded to allow identification of interfering compounds (TFI > 110% control)^26^.

### TR-FRET

Each 15 µL TR-FRET reaction in 384-well black Proxiplate (Perkin Elmer) contained 1 or 10 nM Trx-6His-BCL6 BTB, 300 nM BCOR-A633 and 0.5 or 1 nM anti-6His-Terbium cryptate (Perkin Elmer), in the FP assay buffer (25 mM Hepes pH 8, 100 mM NaCl, 0.05% (v/v) Tween20, 0.5 mM TCEP), supplemented with 0.05% (w/v) bovine serum albumin. Test compounds in DMSO or DMSO alone were added to the wells using and Echo 550 acoustic dispenser to give the appropriate test concentration in 0.7% v/v DMSO final. After a 2 h incubation at room temperature, the plates were read on an Envision plate reader or a Pherastar FSX plate reader with 337 nm laser excitation, a first emission filter at 665 nm and a second emission filter at 615 or 620 nm. Data were normalised and IC_50_ determined as described above for the FP assay.

### Thermal shift assay

Thermal shift assays were performed in 4titude FrameStar (Part No. 4ti-0385) 384 well skirted PCR plates: each well contained 10 µL of sodium acetate pH 6 buffer, 100 mM NaCl, 0.5 mM TCEP, 10 µM BCL6 BTB and 20 × Sypro Orange dye (Life Technologies), with or without inhibitor compound (100–400 µM) at 4% (v/v) final DMSO concentration. Plates were heated on a Biorad CFX384 real time thermocycler with a temperature gradient from 10 to 95 °C with a 0.5 °C increment, 0.15 s/cycle. Data were processed using Vortex (Dotmatics): a shift of the BCL6 BTB melting temperature (ΔT_m_) of + 1 °C or more was classed as indicative of BCL6 compound binding.

### Solubility measurement

Solubility measurements by quantitative ^1^H-NMR or HPLC method were performed as described previously^15^.

### Surface plasmon resonance

All surface plasmon resonance (SPR) experiments were carried out on a Biacore T200 (Cytiva) and amine coupling chemistry was used to immobilise the protein on a research grade CM5 sensor chip. The running buffer consisted of 100 mM sodium acetate, 100 mM sodium chloride, and 1 mM TCEP at pH 6.0. The chip’s surface was activated for 10 min using a 1:1 mixture of 100 mM N-hydroxysuccinimide and 400 mM 1-ethyl-3-(3-dimethylaminopropyl)-carbodiimide. BCL6 BTB protein was injected for 20 min at a concentration of 100 µg/mL in a 10 mM sodium acetate buffer (pH 5.5). Finally, the surface was blocked via an injection of 1 M ethanolamine pH 8.5 for 7 min. The flow rate was maintained at 10 µL/min for all the above procedures and ~ 10,000 response units (RU) of BCL6 was immobilised on the chip. Flow cell one was left unmodified as the reference surface.

Following protein immobilisation, the running buffer was changed to 100 mM sodium acetate, 100 mM sodium chloride, 1 mM TCEP, 0.05% Tween20 (v/v), 5% DMSO at pH 6.0.

All compound handling was performed on an ECHO 550 acoustic liquid dispenser and compounds were added to 384-well polypropylene V-bottomed plates (Greiner), which became the sample plates for the SPR. For K_D_ determinations, six to eight-point concentration range (25–500 µM) was generated by dispensing 0.8 µL of each compound, to which 79.2 µL of running buffer with 4% DMSO was added. The flow rate was 30 µL/min, the injection time for the samples was 60 s, and the dissociation time was 60 s. The surface was not regenerated between sample injections. K_D_ values were calculated from the Langmuir plot under equilibrium conditions using the 1:1 binding model in the Biacore software version 2 (Cytiva).

### Ligand-observed NMR

We developed an indirect method to detect ligand binding to BCL6: in house synthesized fluorinated peptide CF_3_CO-WVIP-NH_2_ (TFA-WVIP-NH_2_) was used as a reporter probe in ligand observed ^19^F-NMR^41^. Each 180 µL ^19^F-NMR peptide displacement assay contained 20 mM HEPES buffer pH 8, 100 mM NaCl, 0.5 mM TCEP, 10% D_2_O, 200 µM reporter TFA-WVIP-NH_2_, with or without 200 µM compound (4% D_6_-DMSO final) and 6 µM BCL6 BTB. Each 180 µL sample reaction was prepared in a 96-well plate and 170 µL transferred to a 3 mm NMR tube (Bruker, Part No. Z112272) using a Gilson liquid handler GX-281. NMR data were collected on a Bruker AVANCEIII 500 MHz spectrometer equipped with a 5 mm BBFO probe. Data were acquired and processed using Bruker Topsin 2.1. The recovery of the reporter NMR signal upon addition of compound indicates competitive binding at the same site as the TFA-WVIP-NH_2_ tetrapeptide on the protein. We quantified the extent of recovery of NMR signal in % of reporter displacement^42^:

$${\text{\% displacement}} = \frac{{{\text{ I}}_{{{\text{reporter(}} + {\text{protein}} + {\text{compound)}}}} - {\text{ I}}_{{{\text{reporter(}} + {\text{protein)}}}} }}{{{\text{ I}}_{{{\text{reporter(}} - {\text{protein)}}}} - {\text{ I}}_{{{\text{reporter(}} + {\text{protein)}}}} }}{\text{X }}100{\text{\% }}$$

Each reporter displacement observed with compound was normalised to the DMSO high and low control samples corresponding, respectively, to the TFA-WVIP-NH_2_ peptide signal detected in the absence and the presence of 6 µM BCL6 BTB protein.

Every experiment included SMRT peptide (amino-acid sequence EGLVATVKEAGRSIHEIPR) as positive control and 4-chlorobenzoic acid as negative control.

### BCL6 crystallisation

The purified BCL6 BTB domain (amino-acid sequence 5–129) was crystallised in the presence of a tetra-peptide with an Ac-WVIP-NH_2_ sequence. A stock solution of WVIP peptide at 100 mM in 100% DMSO was added to a 2 mg/mL solution of purified BCL6 BTB to a final concentration of 1 mM. This mixture was then concentrated to a final protein concentration of 4 mg/mL using a centrifugal concentrator with a 10 KDa molecular weight cut-off. Crystals were grown at 18 °C in hanging drops composed of 2 μL of the BCL6-BTB/WVIP complex and 1 μL of a crystallisation solution consisting of 1 M K_2_HPO_4_, 0.7 M NaH_2_PO_4_, 75 mM sodium acetate buffer pH 4.5 and 2% (v/v) DMSO, equilibrated against 350 μL crystallisation solution. Crystals typically grew in 2 days, and respective compounds were soaked into crystals by adding 0.5 μL compound, dissolved in DMSO to a final concentration of 10 to 200 mM, directly to crystallisation drops, followed by 10–120 min incubation. Crystals were cryo-protected in a solution composed of the crystallisation reagent supplemented with 30% (v/v) ethylene glycol and cryo-cooled in liquid nitrogen.

The purified Flag-TEV-BCL6 BTB 5–129 was crystallised without a tetrapeptide, as the Flag-TEV tag of this construct replaced the WVIP peptide in the crystal packing. The protein was concentrated to a final protein concentration of 10 mg/mL using a centrifugal concentrator with a 10 KDa molecular weight cut-off. Crystals were grown at 18 °C in hanging drops composed of 1.5 μL of the Flag-TEV-BCL6 BTB complex and 1.5 μL of a crystallisation solution consisting of 0.1 M Tris pH 7.5 and 0.80 M Na/K Tartrate, equilibrated against 300 μL crystallisation solution. Crystals typically grew in 2 days, and compounds were soaked as described for the other construct. Crystals were cryo-protected in a solution composed of the crystallisation reagent supplemented with 30% (v/v) ethylene glycol and cryo-cooled in liquid nitrogen.

### Crystallographic data collection, processing, and refinement

X-ray data were collected on a Rigaku FRX-AFC11-VariMax Cu-VHF-Pilatus300K (Institute of Cancer Research, London, UK), at the Diamond Light Source (Harwell campus, Oxfordshire, UK) on beamlines I03 and I04-1, and at the ESRF (Grenoble, France) on beamline ID30A-1. Crystals grown from both BCL6 constructs belonged to the space group P 6_1_ 2 2 and diffracted to a resolution between 1.38 and 2.05 Å. Datasets were integrated with XDS^43^ or DIALS^44^ and scaled and merged with AIMLESS^45^. Structures were solved by molecular replacement using PHASER^46,47^ with a publicly available BCL6 structure (PDB code 3BIM)^9^ with ligand and water molecules removed used as a search model. All protein/ligand structures were manually corrected and rebuilt in COOT^48^ and refined with BUSTER^49^ in iterative cycles. Ligand restraints were generated with GRADE^50^ and MOGUL^51^. The quality of the structures was assessed with MOLPROBITY^52,53^. Data collection and refinement statistics are presented in Table S2.

### InCELL Hunter™

BCL6 cellular target engagement assays were established using the InCELL Hunter™ Target Engagement Kit from DiscoverX-Eurofin. For the assay, the expressed protein comprised the *N*-terminal truncated region of BCL6 BTB domain (HDSD51 plasmid construct encoding amino-acids 14–135) fused to a *C*-terminal ePL tag (enhanced ProLabel, DiscoverX). Constructs were transfected over 24 h in HEK293T cells using Lipofectamine 3000 (ThermoFisher Scientific) and 2.5 × 10^5^ transfected cells / well in phenol red-free OptiMEM medium (ThermoFisher Scientific) were re-plated in 384 assay plates before treating with compounds for 6 h. To complete the assay, cells were incubated for 30 min at room temperature with the InCell Hunter assay reagents mix following the manufacturer’s instructions, before reading the chemiluminescent signal on an Envision (Perkin Elmer) plate reader.

### NanoBRET™

For the cellular NanoBRET assay (Promega UK Ltd, NanoBRET Nano-Glo Detection System, catalogue number N1662), DNA encoding full length BCL6 and SMRT was inserted into pFC32K NanoLuc and pFC14K HaloTag vectors (Promega Ltd) to produce the respective *C*-terminal tagged fusion proteins BCL6-nanoLuc and SMRT-HaloTag. HEK293T cells were plated (5 × 10^5^) in T75 tissue culture flask and bulk transfected 48 h later with Fugene 6 (Promega catalogue number E2691) reagent and 18 µg total DNA plasmids encoding the BCL6-nanoLuc donor and SMRT-HaloTag acceptor at a donor:acceptor DNA ratio of 1:25. At 24 h post-transfection, HEK293T cells were collected and stored in liquid nitrogen in 90% (v/v) FBS (PAN Biotech UK) and 10% (v/v) DMSO. At the time of assay, compounds (100nL/well) and NanoBRET 618 ligand (10nL of 1 mg/ml stock solution per well) were dispensed in a dry 384-well NUNC white assay plate (ThermoScientific NUNC cat. #10080681) using Echo550 acoustic dispensing (Beckman Coulter). Frozen transfected HEK293T cells were thawed, centrifuged, and freezing medium was replaced by phenol red-free OptiMEM + 4% (v/v) FBS (ThermoFisher- Gibco Opti-MEM™ I Reduced Serum Medium, No Phenol Red). The cell density was adjusted to 3 × 10^5^ cells/ml and 20 µL cells (~ 6000 cells) were plated in each well containing test compounds (0.0125–50 µM) in DMSO or DMSO alone and 0.5 µg/ml NanoBRET 618 fluorescence ligand, in 0.55% (v/v) DMSO final concentration. Cells were incubated for 6 h at 37 °C/5% CO_2_ followed by addition of the NanoBRET furimazine substrate (Promega Ltd) to a final concentration of 10 µM. After a short centrifugation, the plates were read on an Envision (Perkin Elmer) plate reader equipped with a LUM/D600 Dual mirror, Lum 450 nm/40 bandpass and D605 nm longpass filters, or on a Pherastar FSX (BMG Labtech) plate reader equipped with a LUM module 610 nm-LP and 450 nm/80 bandpass. The % inhibition at each test concentration was calculated by normalising the BRET acceptor:donor ratio to the appropriate high and low controls. The compound IC_50_ values were determined using Graphpad Prism 6.0 or Dotmatics software by fitting the normalised data to a sigmoidal four-parameter logistic fit equation.

### PAMPA

Passive diffusion permeability was measured using a parallel artificial membrane permeability assay (PAMPA). The assay used an artificial membrane consisting of 2% (v/v) phosphatidyl choline in dodecane (Sigma Aldrich, Dorset, UK). The donor plate was a MultiScreen-IP Plate with a 0.45 µm hydrophobe Immobilon-P membrane (Millipore, UK) and the acceptor plate was a MultiScreen 96-well transport receiver plate (Millipore, UK). The permeability of test compound (10 µM) was measured at pH 7.4 in buffer containing 1% (w/v) Bovine Serum Albumin (Sigma Aldrich, Dorset, UK) following a 16 h incubation at 30 °C. After transfer and centrifugation, sample supernatants were diluted and analysed using a Waters (Milford, MA, US) TQ-S LC-MS/MS system. Permeability values (cm/s) were calculated using the following equation, where V_D_ and V_A_ are the volumes of donor and acceptor, respectively, and area is the surface area of the membrane x porosity:

$${\text{P}}_{{{\text{app}}}} = {\text{ C x ln }}\left( {{1 }{-} \, \left[ {{\text{Drug}}_{{{\text{ acceptor}}}} } \right] \, / \, \left[ {{\text{Drug}}_{{{\text{ equilibrium}}}} } \right]} \right),{\text{ where}}\;{\text{C}} = \frac{{V_{D} {\text{ x }}V_{A} }}{{\left( {V_{D} + { }V_{A} } \right){\text{ x area x time}}}}$$

### Peptides and compounds

Unlabelled peptides BCOR, SMRT and WVIP were obtained from Pepceuticals Ltd; Alexa Fluor®-conjugated BCOR peptide was obtained from Cambridge Research Biochemicals. TFA-WVIP-NH_2_ peptide was purchased from Genecust, France.

The screening campaign was performed on the merged HTS libraries from the Institute of Cancer Research (ICR, Sutton) and the Therapeutic Discovery Laboratory (TDL, Cambridge).

Preparation of chemical compounds, except the oxindole-based compound below, has been described previously^14,15^.

### Preparation of isopropyl 7-((1-methyl-2-oxoindolin-5-yl)amino)pyrazolo[1,5-a]pyrimidine-5-carboxylate (compound *25*)

To a mixture of 5-amino-1-methylindolin-2-one (21 mg, 0.13 mmol) and ethyl 7-chloropyrazolo[1,5-a]pyrimidine-5-carboxylate (29 mg, 0.129 mmol) under argon was added 2-Propanol (1 mL) followed by 37% aqueous HCl (3 drops). The resulting mixture was stirred at 100 °C for 24 h then allowed to cool to rt, diluted with EtOAc (10 mL) and concentrated under reduced pressure. Purification by flash column chromatography (80—100% EtOAc in cyclohexane) afforded compound **25** (15.9 mg, 0.044 mmol, 33.6% yield) as a yellow solid. HRMS (ESI + ve): m/z found 366.1549 expected 366.1561 for C_19_H_20_N_5_O_3_^+^ [M + H]^+^. *δ*_H_ (500 MHz,, CDCl_3_) δ 8.17 (d, *J* = 2.4 Hz, 1H), 8.13 (s, 1H), 7.36 (dd, *J* = 8.3, 2.3 Hz, 1H), 7.33 (s, 1H), 6.96 – 6.90 (m, 2H), 6.84 (d, *J* = 2.3 Hz, 1H), 5.36 – 5.24 (heptet, *J* = 6.4 Hz, 1H), 3.63 (s, 2H), 3.29 (s, 3H), 1.44 (d, *J* = 6.4 Hz, 6H).

## Accession codes

Atomic coordinates and structure factors for the crystal structures of BCL6 BTB domain in complex with the WVIP peptide or with compounds **2**, **7**, **10**, **11**, **13**, **14**, **15**, **17**, **18**, **19**, **21** and **22** can be accessed using PDB codes 7ZWN, 7ZWO, 7ZWP, 7ZWQ, 7ZWR, 7ZWS, 7ZWT, 7ZWU, 7ZWV, 7ZWW, 7ZWX, 7ZWY and 7ZWZ, respectively. The authors will release the atomic coordinates and experimental data upon article publication.

## Supplementary Information

Supplementary Information.
