# Supplementary material for: Discovering cell-active BCL6 inhibitors: effectively combining biochemical HTS with multiple biophysical techniques, X-ray crystallography and cell-based assays
Source: Sci Rep. 2022 Nov 3;12:18633. doi: 10.1038/s41598-022-23264-z (PMC9633773; doi:10.1038/s41598-022-23264-z)
Supplement: Supplementary file 1 — Supplementary Information. [file 41598_2022_23264_MOESM1_ESM.pdf]

Supporting Information

Discovering cell-active BCL6 inhibitors: effectively combining biochemical HTS with multiple biophysical techniques, X-ray crystallography and cell-based assays.

Olivier A. Pierrat[1], Manjuan Liu[1], Gavin W. Collie[1,2], Kartika Shetty[1,2], Matthew J. Rodrigues[1,2], Yann-Vaï Le Bihan[1,2], Emma Gunnell[1,2], Craig McAndrew[1], Mark Stubbs[1], Martin G. Rowlands[1], Norhakim Yahya[1], Erald Shehu[1], Rachel Talbot[1], Lisa Pickard[1], Benjamin R. Bellenie[1], Kwai-Ming J. Cheung[1], Ludovic Drouin[1], Paolo Innocenti[1], Hannah Woodward[1], Owen A. Davis[1], Matthew G. Lloyd[1], Ana Varela Rodriguez[1], Rosemary Huckvale[1], Fabio Broccatelli[1], Michael Carter[1], David Galiwango[1], Angela Hayes[1], Florence I. Raynaud[1], Christopher Bryant[1], Steven Whittaker[1], Olivia W. Rossanese[1], Swen Hoelder[1], Rosemary Burke[1] and Rob L. M. van Montfort[1,2,*]

[1] Centre for Cancer Drug Discovery, Division of Cancer Therapeutics, The Institute of Cancer Research, London SM2 5NG, United Kingdom.

[2] Division of Structural Biology, The Institute of Cancer Research, London SW3 6JB, United Kingdom.

*Correspondence and requests for materials should be addressed to R.L.M.v.M. (email: rob.vanmontfort@icr.ac.uk)

KEYWORDS. B-Cell lymphoma 6, SPR, LO-NMR, NanoBRET, X-ray crystallography

Contents:

1) Figure S1: BCOR peptide interaction in the BCL6 fluorescence polarisation assay.
2) Figure S2: Identification of WVIP peptide by LO-NMR.
3) Figure S3: Binding mode of the SMRT, BCOR and WVIP peptides.
4) Figure S4: Binding mode of validated HTS hits.
5) Figure S5: Initial NanoBRET pair selection.
6) Table S1: Validation of 24 primary hits in orthogonal biophysical assays and in X-ray crystallography.
7) Table S2: Crystallographic data collection and refinement statistics.
8) Table S3: Activity of 10 benzimidazolone-based compounds in the InCELL Hunter^{TM} cellular target engagement assay.

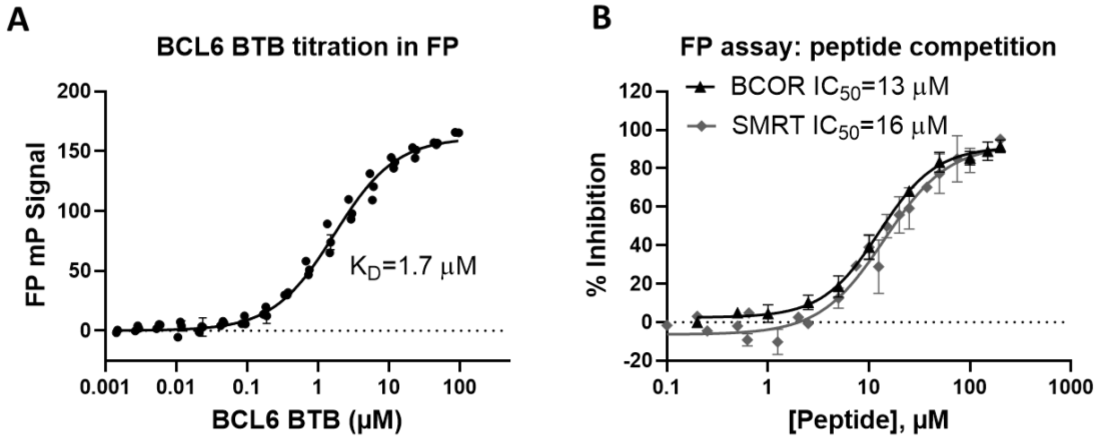

Figure S1: BCOR peptide interaction in the BCL6 fluorescence polarisation assay.

(A) Titration of BCL6 BTB protein and determination of the equilibrium binding constant K_D for the protein-peptide interaction using 10 nM Alexa Fluor 633-conjugated BCOR peptide probe (n=4); (B) Determination of the IC_{50} values of unlabelled peptides BCOR and SMRT in the fluorescence polarisation competition assay with 3 µM BCL6 BTB (n=4).

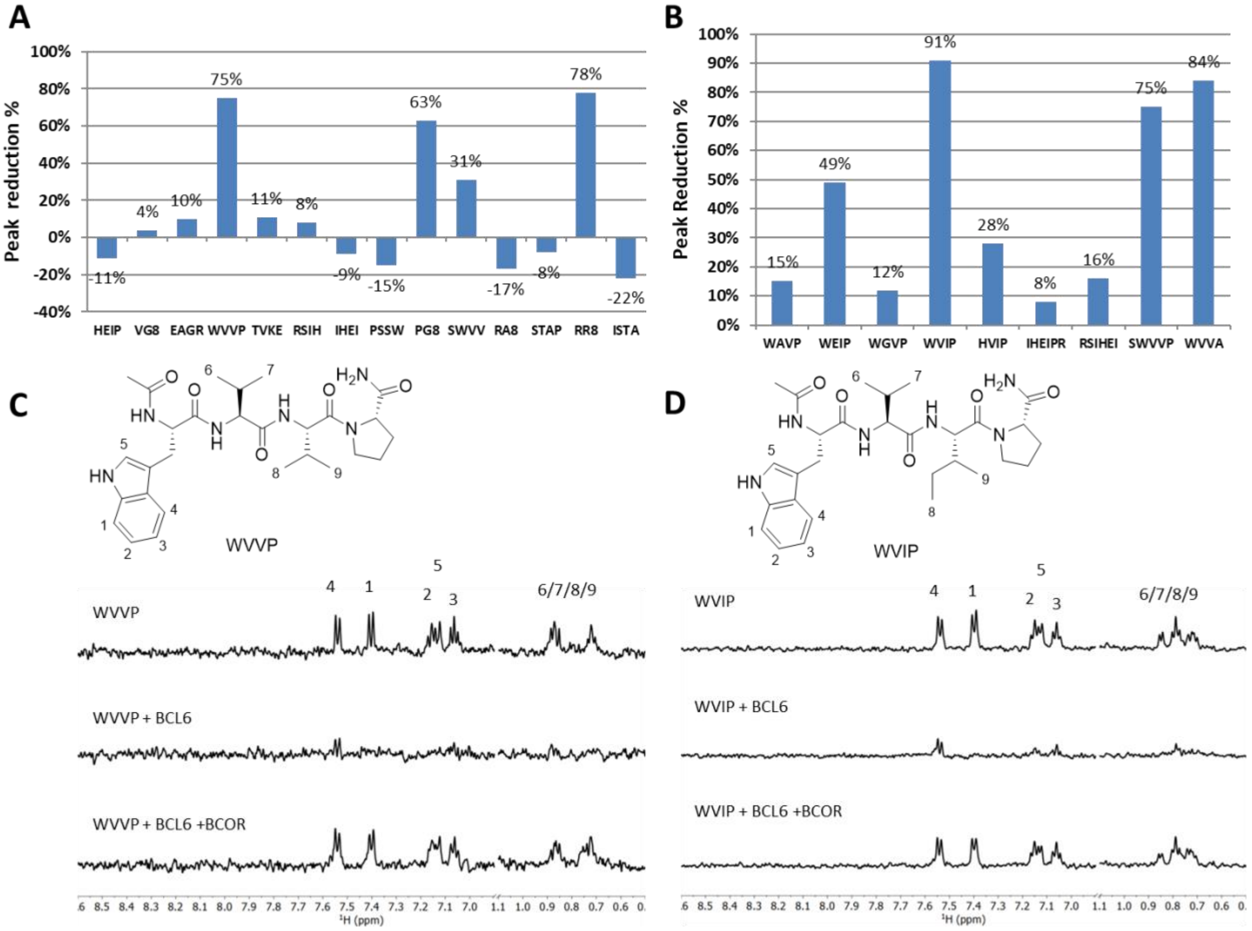

Figure S2: Identification of WVIP peptide by LO-NMR. (A) T2 edited ^{1}H-NMR average peak reduction percentage for peptide fragments originated from BCOR and SMRT BCL6 binding domains in the presence of BCL6 BTB domain. BCOR peptide sequence: RSEIISTAPSSWVVPG; SMRT peptide sequence: LVATVKEAGRSIHEIPR; VG8: VATVKEAG; PG8: PSSWVVPG; RA8: RSEIISTA; RR8: RSIHEIPR; (B) T2 edited ^{1}H-NMR average peak reduction percentage for peptides derived from the WVVP tetrapeptide; (C,D) T2 edited ^{1}H-NMR spectra of WVVP and WVIP showing both peptides binding to the BCOR BCL6 binding site. Key ^{1}H-NMR signal assignments are numbered on the spectra in reference to the proton numbers marked on the structures. The WVVP (C) and WVIP (D) signals were reduced upon binding to BCL6 BTB (top and middle traces) but recovered after competition by BCOR (bottom traces), indicating overlapping binding sites for the short and long peptides.

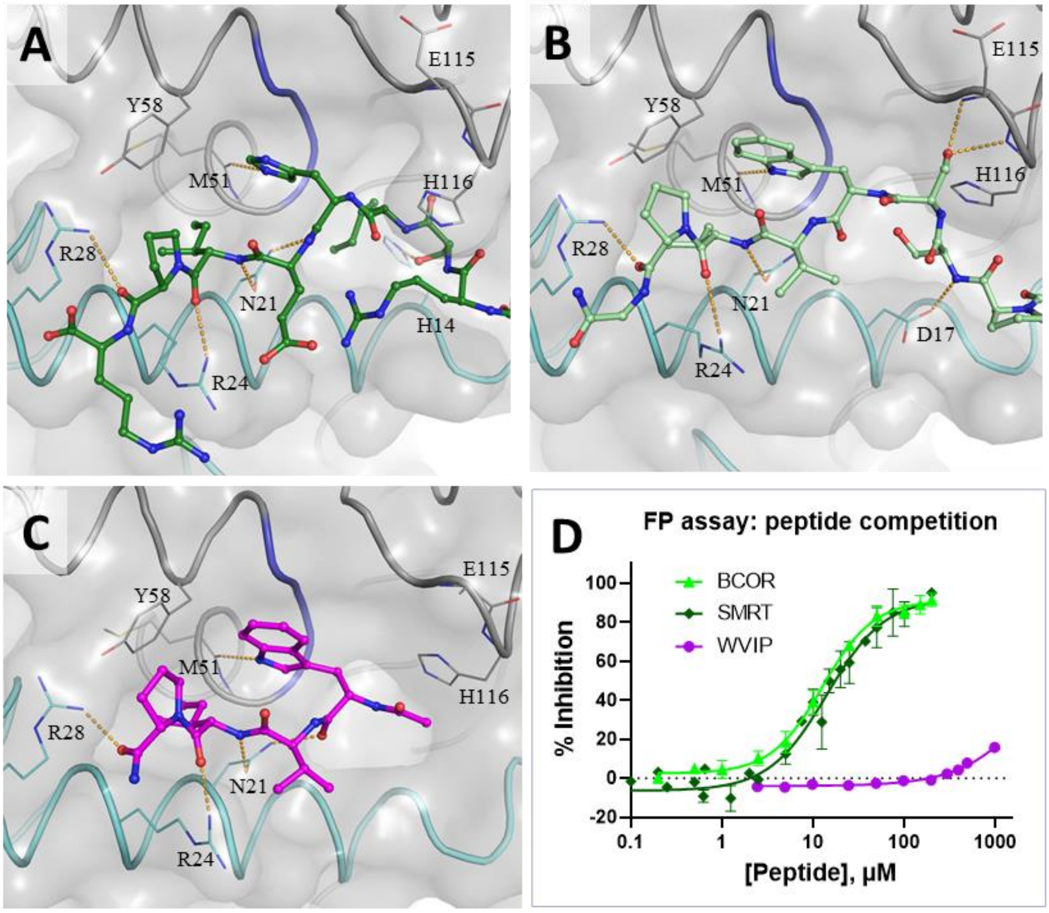

Figure S3: Binding mode of the SMRT, BCOR and WVIP peptides.

(A) Close-up of the binding of the SMRT peptide (dark green, PDB 1R2B), (B) BCOR peptide (light green, PDB 3BIM) and (C) WVIP peptide (magenta) to the BCL6 BTB domain dimer. The colour scheme and surface representation of the BCL6 dimer are the same as in Figure 4. The protein backbone is shown as a ribbon, with residues 53-55 highlighted in blue. Key BCL6 residues are shown as lines and are labelled. Peptides are shown as ball and sticks, and H-bonds as yellow dashed lines. (D) Graph showing the potency of the SMRT, BCOR and WVIP peptides in the FP assay. The potency of the WVIP peptide is weak (16% inhibition at 980 µM, n=4) compared with longer BCOR (IC_{50} = 13 µM (n=4)) and SMRT (IC_{50} = 16 µM (n=4)) peptides.

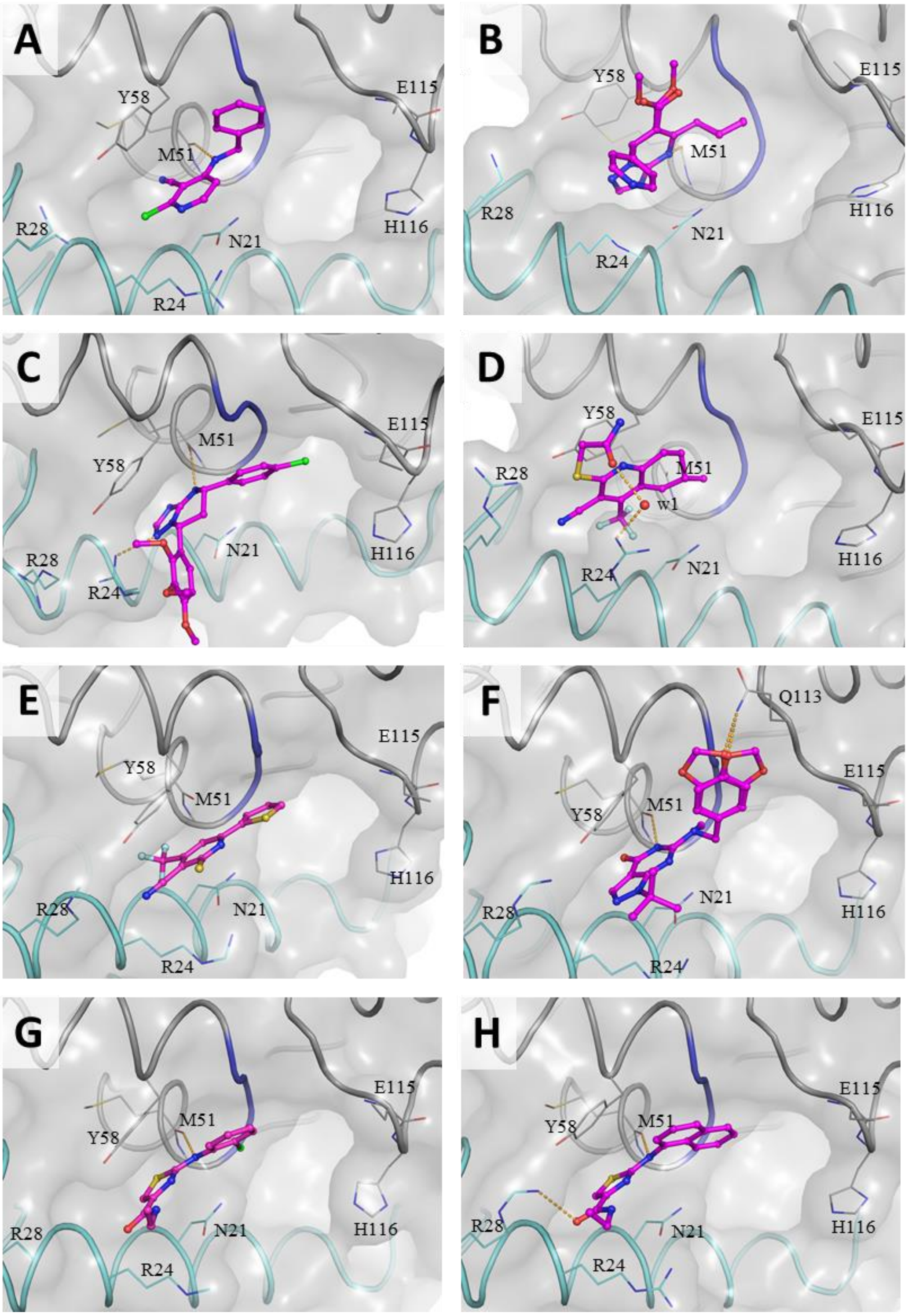

A
Y58
M51
E115
R28
N21
H116
R24
B
Y58
E115
M51
R28
H116
N21
R24
C
M51
E115
Y58
R28
N21
H116
R24
D
Y58
E115
R28
M51
w1
H116
R24
N21
E
E115
Y58
M51
H116
R28
N21
R24
F
Q113
E115
Y58
M51
N21
R28
H116
R24
G
E115
M51
Y58
R28
N21
H116
R24
H
E115
Y58
M51
R28
N21
H116
R24

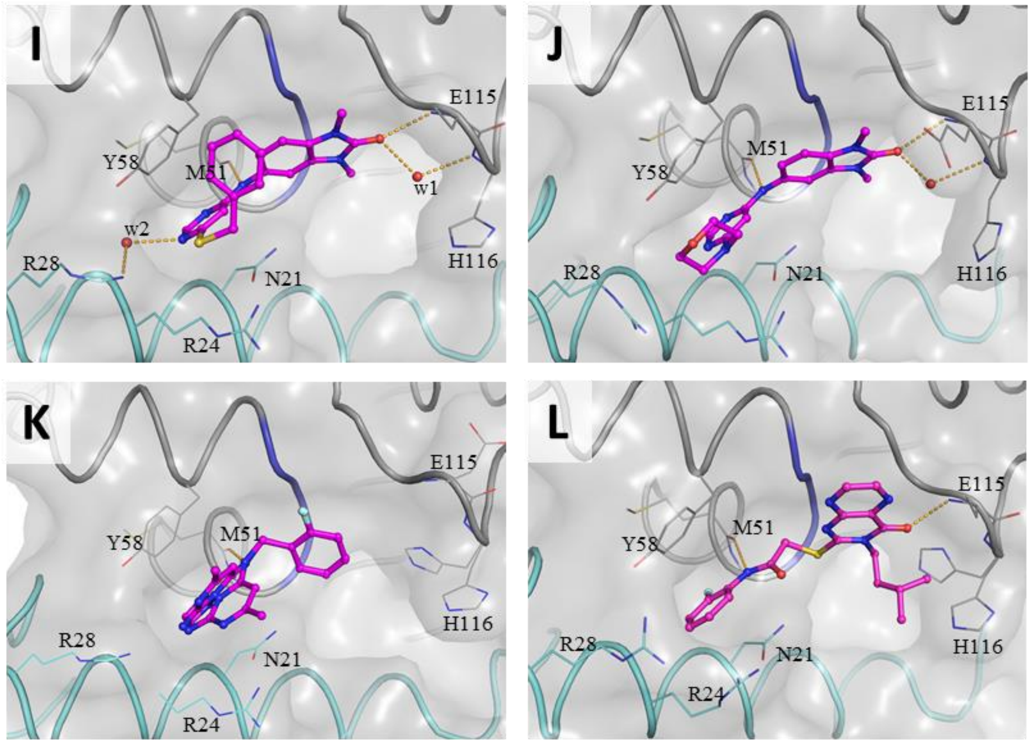

Figure S4: Binding mode of validated HTS hits.

(A) **21**. (B) **7**. (C) **2**. (D) **11**. (E) **13**. (F) **19**. (G) **14**. (H) **15**. (I) **17**. (J) **18**. (K) **10**. (L) **22**. The surface of BCL6 dimer is shown in grey, with the two individual monomers coloured grey and cyan. The protein backbone is shown as a ribbon, with residues 53-55 highlighted in blue. Key BCL6 residues are shown as lines. Compounds are shown as magenta ball and sticks, and H-bonds as yellow dashed lines.

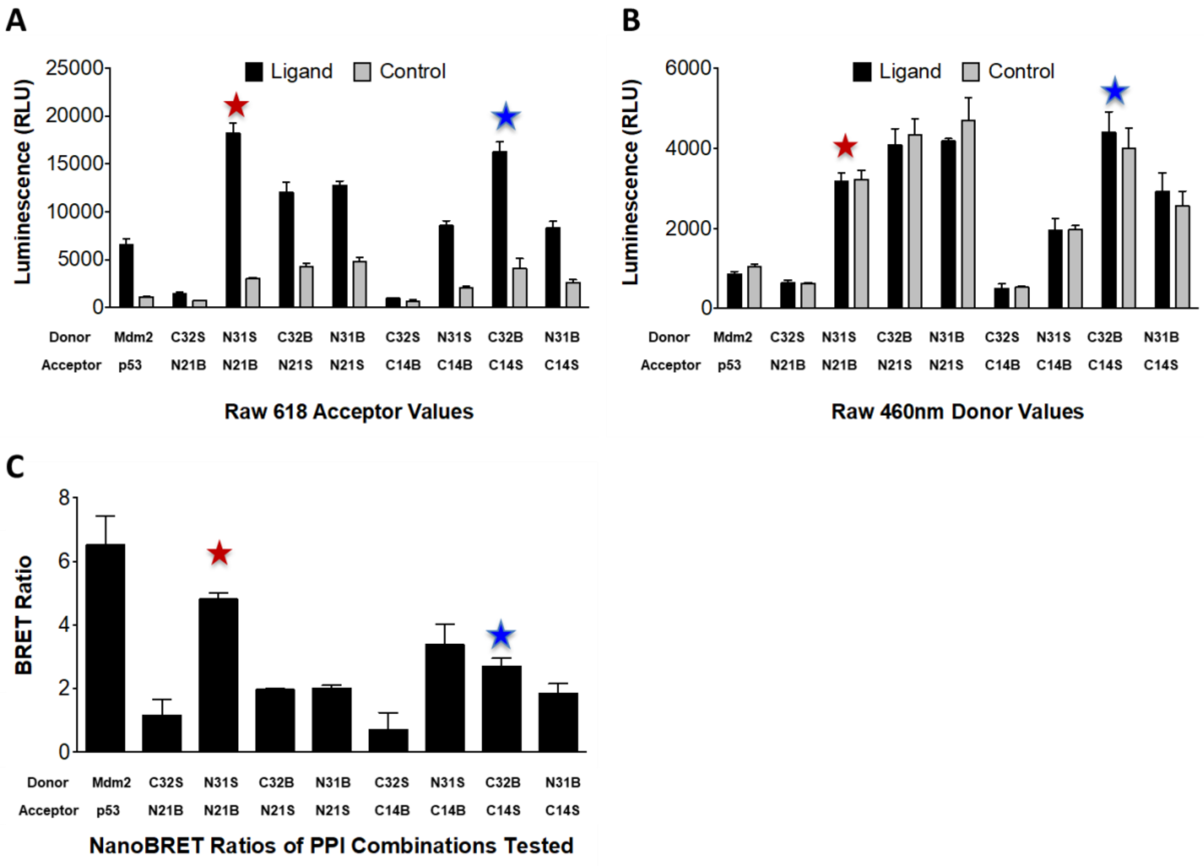

Figure S5: Initial NanoBRET pair selection. Full length BCL6 and SMRT proteins were cloned in various NanoBRET entry vectors, pFC14K.HaloTag, pFC21K.HaloTag, pFC31K.NanoLuc and pFC32K.NanoLuc. Each protein was tagged N-terminally or C-terminally with either the donor NanoLuc Tag or the acceptor HaloTag. Each donor or acceptor listed on the horizontal axis of the graphs is identified by a letter/number combination in which the first letter indicates the position of the tag ("N" for N-terminal and "C" for C-terminal), the following number refers to the nanoBRET entry vector, and the last letter indicates the protein name ("B" for BCL6 and "S" for SMRT). The first NanoBRET donor acceptor pair in each graph is the MDM2/p53 positive control. (A, B) The raw acceptor and donor signals, with or without HaloTag 618 ligand, measured for eight BCL6/SMRT pair configurations. (C) NanoBRET ratio of acceptor over donor signals. For inhibitor testing, we selected two configurations where the donor and acceptor are swapped between the two proteins and for which BRET ratios

and raw signals are at their highest. Between these two, BCL6 was either tagged in N-terminal with the acceptor HaloTag (red star, HaloTag.BCL6) or in C-terminal with the donor NanoLuc Tag (blue star, BCL6.NanoLuc).

Structure	ID No	Cluster / Core chemical scaffold	Sol. (µM)[1]	FP IC_{50} Geo-metric Mean (µM)[2]	FP N	FP pIC_{50} Mean	FP pIC_{50} SD	TSA ΔT_m (ºC)[3]	SPR K_D (µM)[4]	^{19}F LO-NMR, % peptide displaced[5]	X-Ray
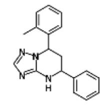	1	1a/ triazolo-pyrimidine	71	197.32	1	3.70	0	0-1	Fail	61 ± 4	ND
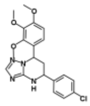	2	1a/ triazolo-pyrimidine	91	201.79	5	3.70	0.04	0.5-1	558	52 ± 3	+
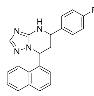	3	1a/ triazolo-pyrimidine	64	101.84	2	3.99	0.25	0.5-2	Fail	54 ± 1	ND
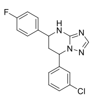	4	1a/ triazolo-pyrimidine	<30	168.09	2	3.77	0.04	0.5-1.5	ND	ND	ND
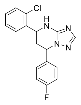	5	1a/ triazolo-pyrimidine	<30	194.29	3	3.71	0.26	0	ND	ND	ND
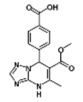	6	1b/ triazolo-pyrimidine	538	702.6	4	3.15	0.01	0.5-1	659	33.5 ± 1	ND
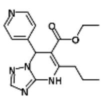	7	1b/ triazolo-pyrimidine	476	95.53	6	4.02	0.04	0.5-3	168	82.5 ± 5	+
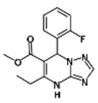	8	1b/ triazolo-pyrimidine	70	218.72	3	3.66	0.10	0.5-2	Fail	ND	ND
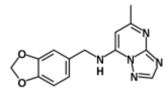	9	1c/ triazolo-pyrimidine	96	476.87	4	3.32	0.03	0.5-1.5	Fail	49.5 ± 9	ND
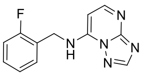	10	1c/ triazolo-pyrimidine	486	464.78	5	3.33	0.12	0	728	32 ± 8	+
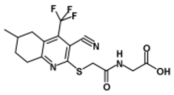	11	2/cyano-trifluoro methyl-pyridine	466	78.41	3	4.11	0.05	1-3.5	412	69.5 ± 8	+
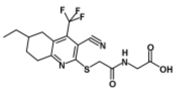	12	2/ cyano-trifluoro methyl-pyridine	494	78.71	4	4.10	0.17	1.5-3.5	1040	75 ± 7	ND
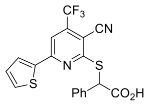	13	2/ cyano-trifluoro methyl-pyridine	313	229.73	1	3.64	0.00	0-2.5	672	102 ± 1	+
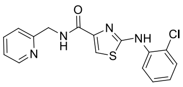	14	3/ thiazole	188	183.48	3	3.74	0.23	1-2.5	302	78 ± 23	+
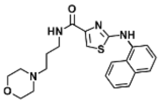	15	3/ thiazole	83	149.51	6	3.83	0.21	0.5-1.5	553	70 ± 1	+

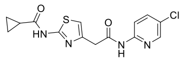	16	3/ thiazole	<30	425.76	3	3.37	0.06	0.5	Fail	36 ± 4	ND
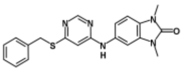	17	4/ pyrimidine-benz-imidazolone	39	70.16	3	4.15	0.18	0.5-1.5	49	100 ± 1	+
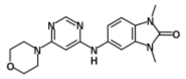	18	4/ pyrimidine-benz-imidazolone	55	>980 (34%)	5	n/a	n/a	0-0.5	ND	ND	+
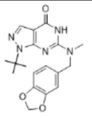	19	5/ pyrazolo-pyrimidinone	21*	191.17	3	3.72	0.08	0	Fail	ND	+
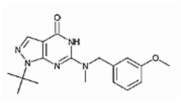	20	5/ pyrazolo-pyrimidinone	<30	666.49	1	3.18	0	0	ND	ND	ND
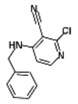	21	S1/ cyano-chloro-pyridine	72	120.04	6	3.92	0.08	1-2	81	63 ± 7	+
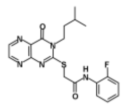	22	S2/ Pteridinone	163	353.38	3	3.45	0.213 6	0.5-1	1190	23 ± 11	+
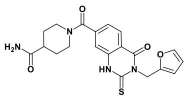	23	S3/ quinazoline-dione	<30	77.18	4	4.11	0.14	0.5	ND	ND	ND
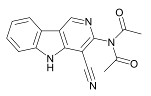	24	S4/ Cyano-pyridoindol	92	471.93	6	3.33	0.04	0	Fail	21 ± 2	Fail

Table S1: Validation of 24 primary hits in orthogonal biophysical assays and in X-ray crystallography. The hits were clustered by chemical structure similarity. [1]NMR kinetic solubility at a final compound concentration of 500 µM in 20 mM HEPES buffer pH 8 containing 2% (v/v) or 5% (v/v) DMSO, or where * is shown, by HPLC at pH 7.4, in phosphate buffered saline (PBS) containing 1% (v/v) DMSO. [2] IC_{50} values representing the geometric mean, n=1-6. [3] Thermal shifts measured at 200 and 400 µM compound concentration – a compound was classed as a binder if $\Delta T_m \geq 1$ °C. [4]A SPR result was classed as a fail when an unsaturating curve, poor quality sensorgram, or a ratio of Experimental Rmax over Theoretical Rmax < 0.5 or > 3 was observed. [5]A compound was defined as a hit in ^{19}F LO-NMR if it showed a minimum displacement of the TFA-WVIP-NH_2 peptide of 30% (mean ± SD, n=2).

Table S2: Crystallographic data collection and refinement statistics.

Crystal system	BCL6/WVIP	BCL6/WVIP	BCL6/WVIP
Ligand	**WVIP peptide**	**Compound 2**	**Compound 7**
PDB code	7ZWN	7ZWO	7ZWP
Crystal			
Space group	P 6_1 2 2	P 6_1 2 2	P 6_1 2 2
Unit cell dimensions (a/b/c in Å)	66.45/66.45/151.20	67.19/67.19/165.74	67.27/67.27/164.40
Unit cell angles (α/β/γ in °)	90/90/120	90/90/120	90/90/120
Data collection and processing			
Beamline	In-house Rigaku	ESRF ID30A-1	DLS I03
Wavelength (Å)	1.5419	0.9660	0.9763
Integration program	XDS	XDS	XDS
Reduction program	AIMLESS	AIMLESS	AIMLESS
Resolution range	45.79 – 2.05	47.62 – 1.39	47.53 – 1.85
Number of unique reflections [a]	13158 (986)	45535 (4374)	19548 (1165)
Completeness [a]	100 (99.9)	100 (100)	99.8 (99.5)
Redundancy [a]	19.4 (10.6)	18.8 (19.3)	11.4 (12.1)
R_{merge} (%) [a]	17.2 (119.3)	12.4 (232.5)	22.9 (299)
I/σ(I) [a]	12.8 (1.9)	14.5 (1.5)	7.7 (1)
$CC_{1/2}$ [a, b]	0.998 (0.666)	0.999 (0.558)	0.994 (0.422)
Refinement			
Program	BUSTER	BUSTER	BUSTER
R_{work} (%)	17.34	16.72	20.08
R_{free} (%)	21.28	18.26	22.71
Number of residues	136	131	131
Number of water molecules	116	194	132
Average B-factor (Å^2)	32.23	25.10	31.11
Ramachandran favoured (%)	96.88	96.83	96.62
Ramachandran outliers (%)	0	0	0
RMSD bonds (Å)	0.014	0.014	0.014
RMSD angles (°)	1.577	1.568	1.570

[a] Values in parentheses are for the highest resolution shell.

[b] Half-dataset correlation coefficient, see: Karplus, P. A.; Diederichs, K. Linking crystallographic model and data quality. *Science* **2012**, *336*, 1030−1033.

Crystal system	Flag-TEV-BCL6	BCL6/WVIP	Flag-TEV-BCL6
Ligand	**Compound 10**	**Compound 11**	**Compound 14**
PDB code	7ZWQ	7ZWR	7ZWT
Crystal			
Space group	P 6_1 2 2	P 6_1 2 2	P 6_1 2 2
Unit cell dimensions (a/b/c in Å)	67.16/67.16/165.13	67.27/67.27/164.47	67.61/67.61/166.07
Unit cell angles (α/β/γ in °)	90/90/120	90/90/120	90/90/120
Data collection and processing			
Beamline	DLS I04-1	ESRF ID30A-1	DLS I03
Wavelength (Å)	0.9159	0.9660	0.9762
Integration program	DIALS	XDS	XDS
Reduction program	AIMLESS	AIMLESS	AIMLESS
Resolution range	47.55 – 1.65	47.54 – 1.47	55.22 – 1.94
Number of unique reflections [a]	27423 (1330)	38445 (3666)	16240 (2472)
Completeness [a]	100 (100)	100 (100)	93.2 (99.9)
Redundancy [a]	12 (10)	19 (18.3)	8.4 (5.0)
R_{merge} (%) [a]	6.2 (290.3)	10.3 (350.7)	17.9 (78.5)
I/σ(I) [a]	15.8 (0.8)	16.6 (1.0)	6.6 (1.5)
$CC_{1/2}$ [a, b]	0.999 (0.343)	0.999 (0.424)	0.992 (0.499)
Refinement			
Program	BUSTER	BUSTER	BUSTER
R_{work} (%)	18.34	17.07	21.86
R_{free} (%)	20.67	19.03	25.07
Number of residues	131	130	131
Number of water molecules	123	165	98
Average B-factor (Å^2)	45.53	30.64	40.18
Ramachandran favoured (%)	99.22	97.60	97.67
Ramachandran outliers (%)	0	0	0
RMSD bonds (Å)	0.015	0.014	0.014
RMSD angles (°)	1.573	1.510	1.670

[a] Values in parentheses are for the highest resolution shell.

[b] Half-dataset correlation coefficient, see: Karplus, P. A.; Diederichs, K. Linking crystallographic model and data quality. *Science* **2012**, *336*, 1030−1033.

Crystal system	BCL6/WVIP	Flag-TEV-BCL6	BCL6/WVIP
Ligand	**Compound 17**	**Compound 18**	**Compound 21**
PDB code	7ZWV	7ZWW	7ZWY
Crystal			
Space group	P 6_1 2 2	P 6_1 2 2	P 6_1 2 2
Unit cell dimensions (a/b/c in Å)	67.23/67.23/165.20	67.60/67.60/166.70	67.62/67.62/164.73
Unit cell angles (α/β/γ in °)	90/90/120	90/90/120	90/90/120
Data collection and processing			
Beamline	ESRF ID30A-1	DLS I04-1	DLS I03
Wavelength (Å)	0.9660	0.9159	0.9763
Integration program	XDS	DIALS	XDS
Reduction program	AIMLESS	AIMLESS	AIMLESS
Resolution range	47.59 – 1.52	47.91 – 1.67	47.73 – 1.65
Number of unique reflections [a]	34987 (3345)	27141 (1374)	27702 (1290)
Completeness [a]	100 (100)	100 (100)	99.8 (97.8)
Redundancy [a]	19 (18.8)	12 (10.2)	34.7 (18.9)
R_{merge} (%) [a]	9.7 (277.1)	4.9 (212.3)	12.6 (231.0)
I/σ(I) [a]	17.4 (1.2)	19.4 (0.9)	22.6 (1.9)
$CC_{1/2}$ [a, b]	1.000 (0.527)	0.999 (0.479)	0.999 (0.527)
Refinement			
Program	BUSTER	BUSTER	BUSTER
R_{work} (%)	16.59	19.02	16.26
R_{free} (%)	16.99	21.54	18.36
Number of residues	130	131	131
Number of water molecules	158	128	205
Average B-factor ($Å^2$)	32.01	45.82	28.74
Ramachandran favoured (%)	96.80	97.67	98.41
Ramachandran outliers (%)	0	0	0
RMSD bonds (Å)	0.015	0.014	0.014
RMSD angles (°)	1.577	1.465	1.505

[a] Values in parentheses are for the highest resolution shell.

[b] Half-dataset correlation coefficient, see: Karplus, P. A.; Diederichs, K. Linking crystallographic model and data quality. *Science* **2012**, *336*, 1030−1033.

Crystal system	BCL6/WVIP	BCL6/WVIP	BCL6/WVIP
Ligand	**Compound 19**	**Compound 22**	**Compound 15**
PDB code	7ZWX	7ZWZ	7ZWU
Crystal			
Space group	P 6_1 2 2	P 6_1 2 2	P 6_1 2 2
Unit cell dimensions (a/b/c in Å)	67.25/67.25/165.15	67.53/67.53/165.52	67.41/67.41/165.13
Unit cell angles (α/β/γ in °)	90/90/120	90/90/120	90/90/120
Data collection and processing			
Beamline	ESRF ID30A-1	ESRF ID30A-1	ESRF ID30A-1
Wavelength (Å)	0.9660	0.9660	0.9660
Integration program	XDS	XDS	XDS
Reduction program	AIMLESS	AIMLESS	AIMLESS
Resolution range	47.59 – 1.38	47.76 – 1.40	47.67 – 1.56
Number of unique reflections [a]	46419 (4460)	44966 (2180)	32153 (1593)
Completeness [a]	100 (100)	100 (100)	98.8 (99.5)
Redundancy [a]	18.9 (19.3)	16.9 (16.3)	11.2 (9.3)
R_{merge} (%) [a]	14.6 (330.9)	10.3 (285.5)	10.0 (87.1)
I/σ(I) [a]	11.3 (1.0)	16.3 (1.4)	14.6 (2.5)
$CC_{1/2}$ [a, b]	0.999 (0.460)	0.998 (0.623)	0.999 (0.381)
Refinement			
Program	BUSTER	BUSTER	BUSTER
R_{work} (%)	17.22	16.71	15.70
R_{free} (%)	18.89	17.45	17.66
Number of residues	131	131	131
Number of water molecules	167	184	164
Average B-factor (Å^2)	27.77	26.59	26.13
Ramachandran favoured (%)	96.83	96	97.62
Ramachandran outliers (%)	0	0	0
RMSD bonds (Å)	0.014	0.014	0.014
RMSD angles (°)	1.555	1.589	1.546

[a] Values in parentheses are for the highest resolution shell.

[b] Half-dataset correlation coefficient, see: Karplus, P. A.; Diederichs, K. Linking crystallographic model and data quality. *Science* **2012**, *336*, 1030−1033.

Crystal system	BCL6/WVIP
Ligand	**Compound 13**
PDB code	7ZWS
Crystal	
Space group	P 6_1 2 2
Unit cell dimensions (a/b/c in Å)	67.11/67.11/164.38
Unit cell angles (α/β/γ in °)	90/90/120
Data collection and processing	
Beamline	ESRF ID30A-1
Wavelength (Å)	0.9660
Integration program	XDS
Reduction program	AIMLESS
Resolution range	58.12 – 1.53
Number of unique reflections [a]	33926 (1628)
Completeness [a]	99.9 (98.6)
Redundancy [a]	8.4 (6.7)
R_{merge} (%) [a]	10.8 (83.5)
I/σ(I) [a]	11.4 (2.2)
$CC_{1/2}$ [a, b]	0.996 (0.335)
Refinement	
Program	BUSTER
R_{work} (%)	16.33
R_{free} (%)	17.79
Number of residues	131
Number of water molecules	154
Average B-factor (Å^2)	28.56
Ramachandran favoured (%)	97.62
Ramachandran outliers (%)	0
RMSD bonds (Å)	0.014
RMSD angles (°)	1.553

[a] Values in parentheses are for the highest resolution shell.

[b] Half-dataset correlation coefficient, see: Karplus, P. A.; Diederichs, K. Linking crystallographic model and data quality. *Science* **2012**, *336*, 1030−1033.

Compound No	InCELL Geomean EC_{50}	InCELL N	InCELL SD
**26**	25.1	2	9.8
**27**	23.7	3	9.3
**28**	27.4	2	2.1
**29**	74.4	2	3.6
**30**	75.0	1	--
**31**	5.0	2	1.8
**32**	9.1	1	--
**33**	7.5	1	--
**34**	75.0	2	2.2
**35**	11.6	2	0.0

Table S3: Activity of 10 benzimidazolone-based compounds in the InCELL Hunter™ cellular target engagement assay. HEK293T cells, transfected with C-terminally ePL-tagged BCL6 BTB 14-135, were incubated with increasing concentrations of compound for 6 hours before luminescence detection. The luminescence signal increased as compound mediated the cellular stabilisation of the enhanced ProLabel (ePL) tagged-BCL6, with EC_{50} values ranging from 5 to 75 µM.
